# Application of Targeted Mass Spectrometry for the Quantification of Sirtuins in the Central Nervous System

**DOI:** 10.1038/srep35391

**Published:** 2016-10-20

**Authors:** T. Jayasena, A. Poljak, N. Braidy, L. Zhong, B. Rowlands, J. Muenchhoff, R. Grant, G. Smythe, C. Teo, M. Raftery, P. Sachdev

**Affiliations:** 1 Centre for Healthy Brain Ageing (CHeBA), School of Psychiatry, University of New South Wales, Sydney, Australia; 2 Bioanalytical Mass Spectrometry Facility, Mark Wainwright Analytical Centre, University of New South Wales, Sydney, Australia; 3 School of Medical Sciences, University of New South Wales, Sydney, Australia; 4 Sydney Medical School, University of Sydney, Sydney, Australia; 5 Minimally Invasive Cancer Centre, Prince of Wales Hospital, Sydney, Australia; 6 Neuropsychiatric Institute, the Prince of Wales Hospital, Sydney, Australia

## Abstract

Sirtuin proteins have a variety of intracellular targets, thereby regulating multiple biological pathways including neurodegeneration. However, relatively little is currently known about the role or expression of the 7 mammalian sirtuins in the central nervous system. Western blotting, PCR and ELISA are the main techniques currently used to measure sirtuin levels. To achieve sufficient sensitivity and selectivity in a multiplex-format, a targeted mass spectrometric assay was developed and validated for the quantification of all seven mammalian sirtuins (SIRT1-7). Quantification of all peptides was by multiple reaction monitoring (MRM) using three mass transitions per protein-specific peptide, two specific peptides for each sirtuin and a stable isotope labelled internal standard. The assay was applied to a variety of samples including cultured brain cells, mammalian brain tissue, CSF and plasma. All sirtuin peptides were detected in the human brain, with SIRT2 being the most abundant. Sirtuins were also detected in human CSF and plasma, and guinea pig and mouse tissues. In conclusion, we have successfully applied MRM mass spectrometry for the detection and quantification of sirtuin proteins in the central nervous system, paving the way for more quantitative and functional studies.

Sirtuins are a class of proteins that possess histone deacetylase or mono-ribosyltransferase activity and play critical roles in cell survival in response to oxidative stress and caloric restriction (CR) regimes^1^. In mammals, seven sirtuins (SIRT1-7) have been identified. All mammalian sirtuins contain a conserved NAD-binding and catalytic domain, but differ in their N and C-terminal domains. They have different specific substrates including histones, transcriptional regulators and enzymes. They localise to cell compartments which regulate cellular structure, metabolism and gene expression, including the cytoskeleton (SIRT2), mitochondria (SIRT3, SIRT4 and SIRT5) and nucleus/nucleolus (SIRT1, SIRT6 and SIRT7), and play important roles in health and disease^1^. SIRT1 is the best characterized and has the broadest substrate specificity. Sirtuins have emerged as critical modulators of metabolic adaptive responses, and their activities have been linked to ageing and multiple diseases, from metabolic abnormalities to neurodegeneration.

Sirtuins can affect reactive oxygen species (ROS) production and promote resistance to their damaging effects. Oxidative stress has been shown to decrease SIRT1 expression in the hippocampus and cortex, possibly by direct degradation by ROS^2^. SIRT1 overexpression prevents oxidative stress-induced apoptosis and increases resistance to oxidative stress through regulation of the FOXO family of forkhead transcription factors^3^. The cytoplasmic sirtuin protein SIRT2, has been shown to increase in response to oxidative stress but promotes cell death through FOXO proteins^4^. SIRT3, a mitochondrial protein, reduces oxidative stress through activation of superoxidase dismutase^5^. SIRT6 and SIRT7, like the founding member of the sirtuin family SIRT1, are nuclear proteins involved in oxidative-stress induced DNA repair through activation of the PARP-1 DNA repair enzyme.

SIRT1 is expressed in the adult brain, in the cortex, hippocampus, cerebellum, and hypothalamus, and in lower levels in the white matter^6^. Among the brain cell types, SIRT1 is predominantly expressed in neurons and viewed as a nuclear protein^6^. The mRNAs for all seven sirtuins have been identified in mouse brain tissue and also neural stem cells^7^. SIRT1, SIRT2 and SIRT3 have also been detected in human serum, and levels were shown to decline with age and were linked to frailty^8^,^9^. SIRT3 is elevated at both the mRNA and protein levels in Alzheimer’s disease (AD) *post mortem* brain tissue compared to controls^10^.

The most common techniques currently utilised for detecting a change in sirtuin levels at the mRNA or protein level are PCR and western blotting, respectively (see Table 1). Other studies have used methods such as immunohistochemistry, surface plasmon resonance and ELISA assays (see Table 1). The majority of these methods are only semi-quantitative with moderate sensitivity, use antibodies which may not have sufficient specificity or detect expression at the mRNA level which may not reflect protein expression. Furthermore, there is no current assay which detects multiple sirtuins simultaneously.

Quantitative expression analysis of mammalian sirtuin proteins (especially SIRT2-7), across cell and tissue types is limited in the current literature. Previous studies have shown an increase in SIRT3 in AD post-mortem brain tissue using western blotting for protein expression and multiplex qPCR to assay SIRT3 mRNA levels^10^. Both SIRT3 protein and mRNA were shown to be significantly elevated in the AD group^10^. Another recent paper detected SIRT1 in human serum samples using western blotting, surface plasmon resonance and ELISA to measure SIRT1 protein levels^8^. SIRT1 declines with age and is more dramatically reduced in MCI and AD patients compared to age matched controls, suggesting that SIRT1 may warrant further investigation as a potential plasma biomarker for AD^8^. SIRT1 and SIRT3 levels in serum were found to be significantly lower in frail subjects as compared to the non-frail^9^. Another study reported a decrease in SIRT1 and SIRT2 mRNA levels using quantitative real time PCR in the primary motor cortex of human post-mortem amyotrophic lateral sclerosis brain tissue^11^.

Mass spectrometry has been used to successfully assay metabolites of SIRT1 activator drugs in plasma and urine^12^, but to date has not been used to quantify protein levels directly. Mass spectrometry has a great advantage over the conventionally used antibody based methods, such as western blotting, in that it offers greater specificity, linearity, reproducibility and typical limits of quantification down to the low fmol range. It also removes some of the antibody specificity issues that are associated with methods such as western blotting and ELISA, as peptides unique to each protein are measured. Targeted MRM based mass spectrometry may provide a more specific and sensitive method to detect and quantify sirtuin expression at the protein level, providing a tool for comprehensive sirtuin protein expression analysis. The aim of this study was to develop a targeted mass spectrometry method using multiple reaction monitoring (MRM) to quantify the seven human sirtuins at the protein level and to apply the assay to CNS biological samples.

## Results

### Peptide standards

**Figure 1:**
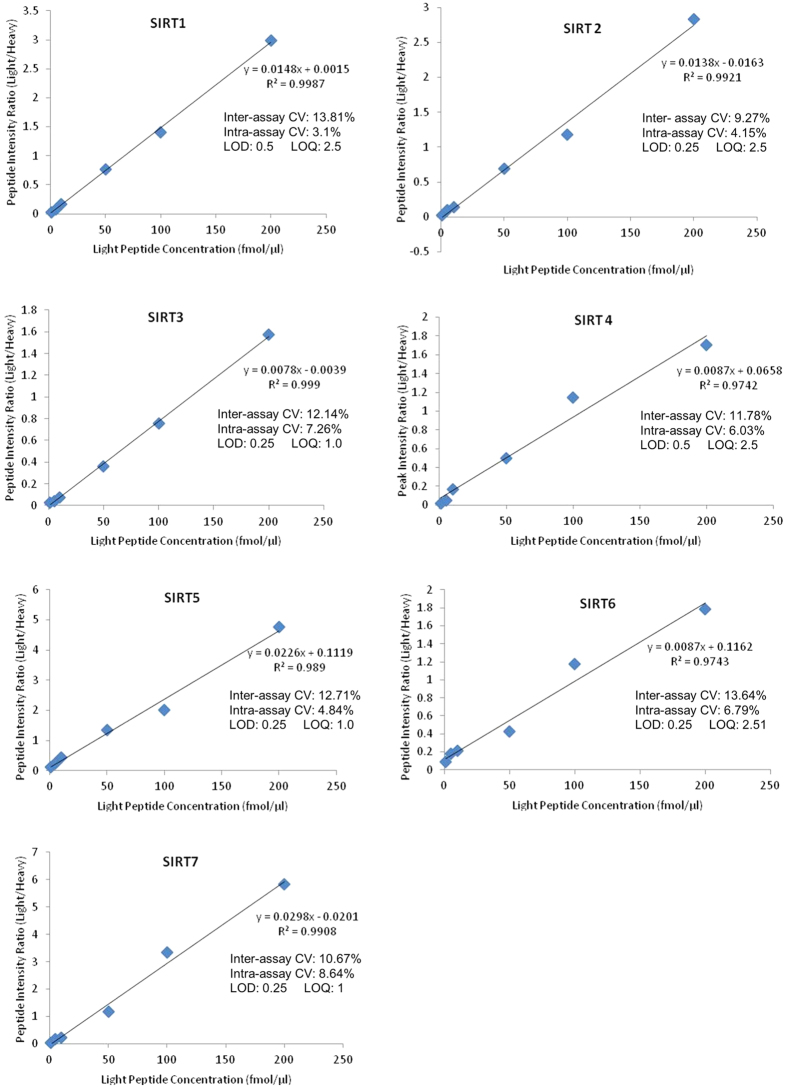
Sirtuin peptide standard curves, variance and limits of detection and quantification. Sirtuin peptide standard curves (average of the peak area ratios for the two peptides, in triplicate for each sirtuin, with heavy peptide spike of 100 fmol/μl). Both inter- and intra-assay variance were calculated for three replicates and the LOD and LOQ are shown in fmol/ul. CVs calculated using peptide peak area ratios (light/heavy) at the 100 fmol/μl peptide concentration level. Individual chromatograms for each of the 14 peptides can be found in Supplementary Figure S1.

Standard curves for sirtuin peptides are shown in Fig. 1. The LOD and LOQ were determined when the signal to noise ratio of the transition with the highest intensity was approximately 3:1 and 10:1 respectively. All peptides had good linearity in the 1–200 fmol/μl range and intra- and inter- assay variance was <10% and less than <14% respectively. Representative chromatograms for each of the 14 peptides are shown in Supplementary Figure S1.

Sirtuin peptide standard curves spiked with buffer only (0.1% formic acid and no gel bit), blank gel bits and gel bits containing Hu6 depleted plasma had very similar regression equations and slopes, indicating modest matrix effects (Supplementary Figure S2). Sirtuin recoveries (from the gel spike experiment, Supplementary Figure S8), relative to sirtuin run in buffer only achieved 61–96% recovery and 58–131% recovery for the 5 μg and 2 μg sirtuin levels respectively (Supplementary Table S1).

### Quantification of sirtuin expression in cells and tissues

**Figure 2:**
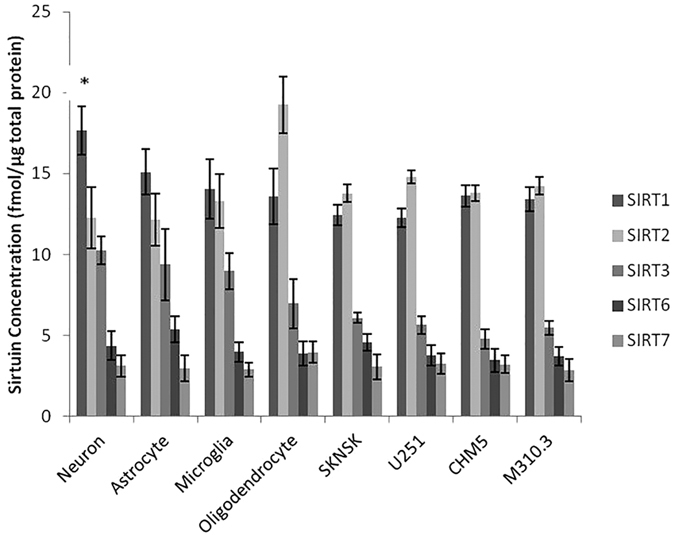
Sirtuin expression in primary cultured brain cells and cell lines. In primary neurons SIRT1 was found to be the most abundant sirtuin (*p < 0.05) compared to other neuronal sirtuins (n = 3). SIRT2 was found to be abundant in primary oligodendrocytes (*p < 0.05) compared to other cell types (n = 3). SIRT1 and SIRT2 were the most abundant in all the cell cultures. SIRT4 and SIRT5 were below the detection limits and only small amounts of SIRT6 and SIRT7 were detected.

**Figure 3:**
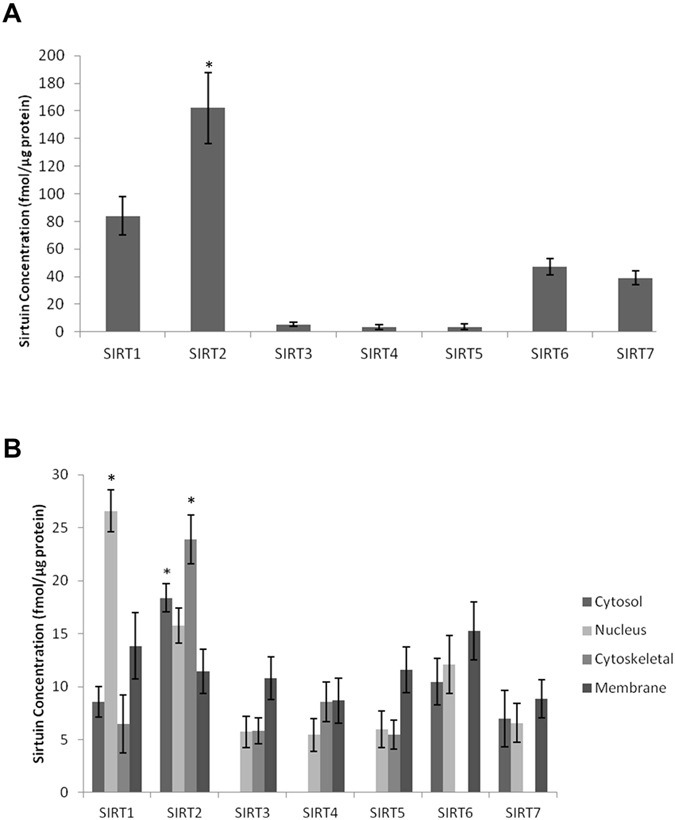
Sirtuin expression in human frontal lobe brain tissue. Sirtuin expression in unfractionated (Panel A) and fractionated (Panel B) human frontal lobe brain tissue. All seven sirtuins were detected in unfractionated human frontal lobe brain tissue with SIRT2 the most abundant (*p < 0.05) compared to all other sirtuins (Panel A). The mitochondrial sirtuins (SIRT3-5) were found to be close to the LOQ, but their signals improved following fractionation of the tissue into cytosolic, nuclear, cytoskeletal and membrane fractions (Panel B). Fractionation showed SIRT1 and SIRT2 expressed in all fractions, whereas SIRT3-5 were below the LOD in the cytosol and SIRT6 and SIRT7 were below the LOD in the cytoskeletal fraction. SIRT1 was most abundant in the nucleus and SIRT2 in the cytoskeletal fraction (*p < 0.05, Panel B). In the cytosol, SIRT2 was the most abundant sirtuin (*p < 0.05), and all sirtuins were captured in the membrane fraction.

In all the human primary brain cell types and human brain cell lines SIRT1, 2, 3, 6 and 7 were detected and quantified (Fig. 2). All seven sirtuins were detected in human control brain tissue, with SIRT2 highly expressed (Fig. 3, Panel A). Detergent fractionation into subcellular groups improved identification of mitochondrial sirtuins (SIRT3–5) (Fig. 3, Panel B).

**Figure 4:**
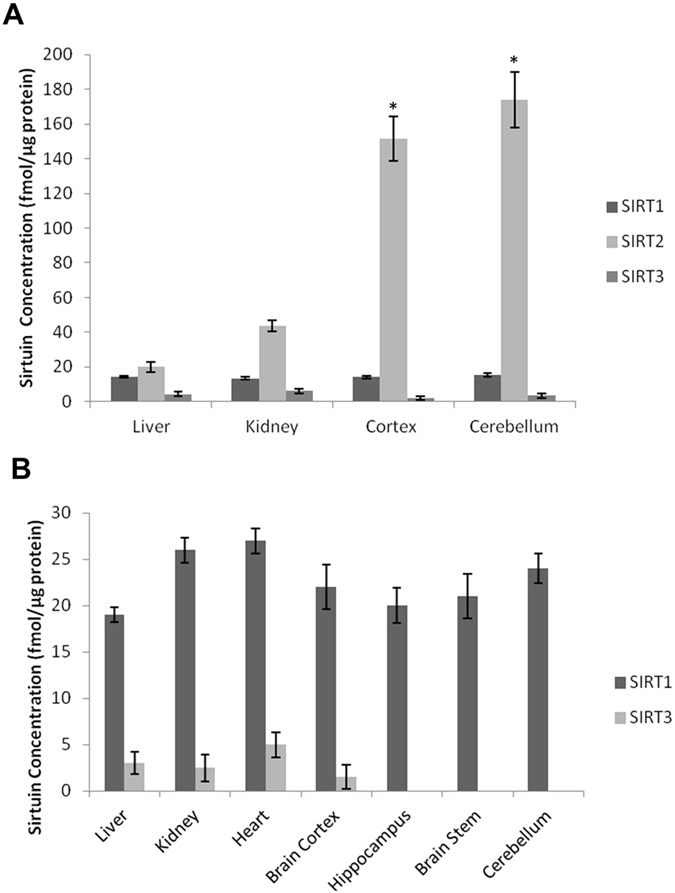
SIRT1-3 protein expression in animal organs. SIRT1-3 protein expression in guinea pig (Panel A) and mouse organs (Panel B). SIRT2 was found to be the most abundant sirtuin in guinea pig, with higher levels expressed in the brain (*p < 0.01, n = 2) compared to liver and kidney. SIRT2 in mouse was not quantified due to peptide sequence difference with the human peptide standards.

We were also able to quantify some sirtuins in guinea pig and mouse tissue due to their common sequences with human sirtuin peptides. SIRT1-3 levels in the guinea pig (Fig. 4, Panel A) and SIRT 1 and 3 levels in mouse organs (Fig. 4, Panel B) were quantified. Furthermore the method could be adapted for all sirtuins from other species with purchase of the appropriate synthetic peptides and their heavy internal standards. Supplementary Table S2 shows the level of homology (or identity) of human SIRT1-7 peptide sequences with mouse and guinea pig. All samples were fractionation on a 1D-SDS-PAGE to reduce sample complexity and improve detection sensitivity. A workflow of the sample preparation procedure can be found in Supplementary Figure S3.

Another important issue is the existence of splice variants or isoforms of the sirtuin proteins. Supplementary Table S3 lists the sequences of all sirtuin isoforms and the location of the two MRM peptides within each sequence. The majority of sirtuin isoforms contain both peptides, and all contain at least one of the two peptide sequences used for quantification in this study.

### Validation of MRM method with established protocols

**Figure 5:**
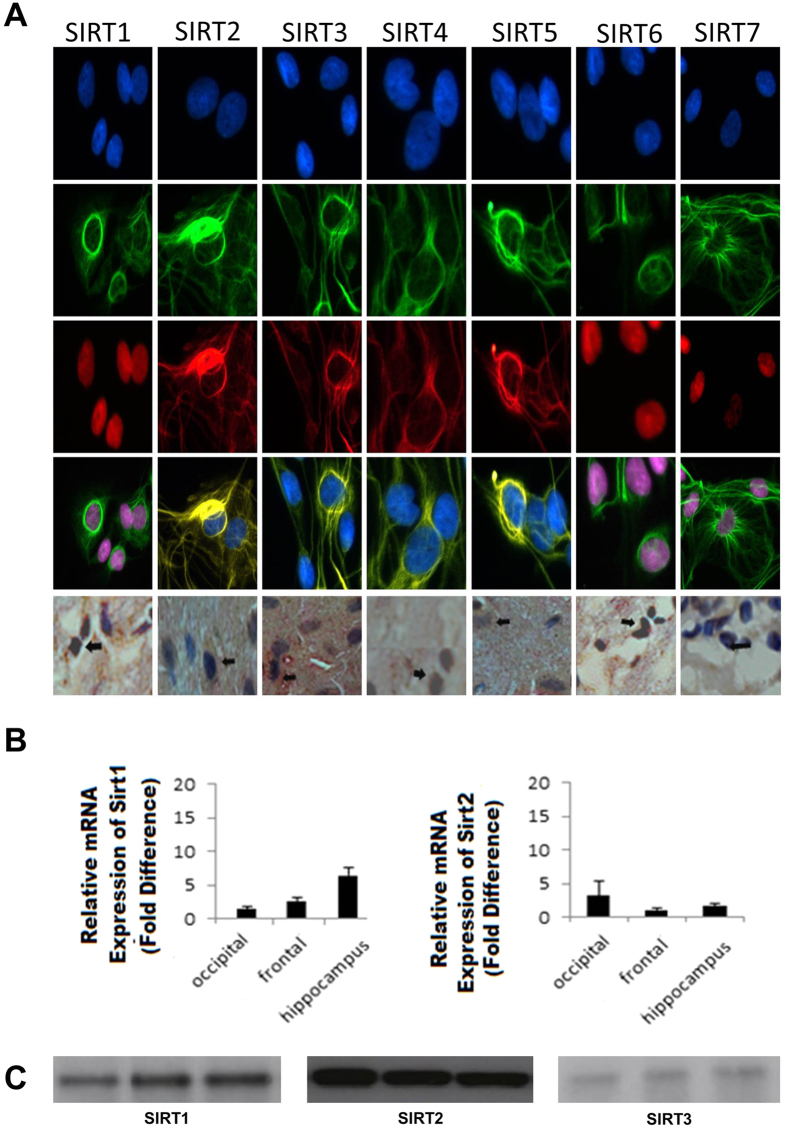
Sirtuin expression in human brain cells and tissue using immunohistochemical staining of astrocytes (Panel A row 1–4) and human frontal lobe brain tissue (Panel A, row 5), PCR of SIRT1 and SIRT2 mRNA in occipital, frontal and hippocampal human control brain (Panel B) and cropped images of western blotting of SIRT1-3 protein expression in human control frontal lobe brain tissue (n = 3) at molecular weights of approx 40 kDa, 50 kDa and 30 kDa respectively (Panel C). Full length blots are presented in Supplementary Figure S4.

Human brain cells and tissue samples were compared with established protocols for sirtuin detection such as immunohistochemical staining, PCR and western blotting. Immunohistochemical staining showed that all seven sirtuins are expressed in astrocytes (Fig. 5, panel A, rows 1–4) and control human frontal lobe brain tissue (Fig. 5, panel A, row 5). PCR identified SIRT1 and SIRT2 mRNA in control human frontal, occipital and hippocampus brain tissue (Fig. 5, panel B). Western blotting confirmed the identification of SIRT1, 2 and 3 in control human frontal lobe brain tissue (Fig. 5, panel C and Supplementary Figure S4).

### Quantification of sirtuins in CSF and immunodepleted plasma

SIRT1 was quantified in the CSF of five control patients and in control plasma immunodepleted of the six most abundant proteins. SIRT1 was the only sirtuin detected and was found to range from 4.30 ± 0.19 to 5.09 ± 0.53 fmol/μg total protein in CSF and 8.68 ± 0.35 fmol/μg total protein in plasma. Individual data can be found in Supplementary Figure S5. SIRT2-7 in CSF and immunodepleted plasma were found to be below the detection limits of the assay. All samples were fractionated by 1D-SDS-PAGE to reduce sample complexity and a workflow of the sample preparation procedure can be found in Supplementary Figure S3.

## Discussion

The rapid expansion of instruments and software in the field of targeted protein quantification by MRM is expected to have vast applications in quantitative protein biochemistry^13^,^14^. Most of the currently used protocols to measure sirtuin changes in the CNS have ultilised methods such as ELISA, surface Plasmon resonance, western blotting and fluorescent staining. Mass spectrometry provides a platform that overcomes some of the limitations of antibody based approaches for protein quantification, in particular providing a level of specificity not available with the other approaches. Isotopically labelled peptide standards allow for quantification of protein levels and also provide the ability to monitor stability of analytes throughout the sample processing steps. This greatly simplifies the development of assays compared with standard immunological formats such as ELISA where well-characterised antibodies are needed. Furthermore MRM facilitates multiplexed analysis, identifying several proteins in a single run, thereby maximising information obtained per sample, minimising assay time while at the same time conserving precious samples. It measures several transitions per quantified protein, thus generating several independent measurements, and with the use of heavy peptide standards allows for generation of standard curves and accurate quantification. This approach has advantages over methods such as western blotting and staining techniques which are semi-quantitative at best and lack the level of structural specificity achieved by mass spectrometry. Our MRM approach is also complementary to PCR which targets mRNA levels only.

Perhaps the major limitation to mass spectrometry based protein quantification is throughput and cost. Immunological assays can be performed in 96 well plate formats and plate readers allow measurement of an entire plate in a single run. Mass spectrometry requires each sample to be run individually with longer run times. However one of the main advantages of mass spectrometry is its ability to specifically and accurately distinguish different isoforms or modified forms of proteins, even in complex samples. The sensitivity of mass spectrometry is very high but limitations to sensitivity of quantitative assays are often caused by the dynamic range of the proteins in the sample. Future developments in mass spectrometer analysers and detectors may help address this limitation. Even so, it is probable that improved sample purification/fractionation procedures will continue to be a vital element of the most challenging and complex proteomics problems, and to achieve the required sensitivity for robust quantification. For example in this study all samples were fractionation on a 1D-SDS-PAGE gel and gel bands excised and tryptic digested prior to MRM analysis to reduce sample complexity. A workflow of the sample preparation procedure can be found in Supplementary Figure S3.

MRM mass spectrometry was successfully utilised in this study to quantify seven mammalian sirtuins in human brain cells, tissues and fluid. Furthermore the method can be used to measure sirtuin expression in animal tissues, such as mouse and guinea pig, in cases where peptide sequences are identical to the human standards. In line with previous data, our results affirm that there is significant divergence in abundance amongst members of the sirtuin family of proteins in the brain. We report SIRT1 and SIRT2 to be the most abundant sirtuins in cultured brain cells with SIRT1 highest in neurons and SIRT2 highest in oligodendrocytes, validating previous studies showing SIRT2 to be highly expressed in oligodendrocytes^15^. Our study and others have also found SIRT2 expressed in neurons and glial cells^16^,^17^. Further validation with higher sample numbers and across a wider range of cell lines may help elucidate differences between cell types for the lower abundant sirtuins.

We found SIRT2 to be the most abundant sirtuin in the adult human frontal lobe and cortex and cerebellum homogenates from the guinea pig. SIRT2 is known to co-localise with microtubules and functions as a tubulin-deacetylase. It is a suppressor of microglial activation and brain inflammation, with reduced levels of SIRT2 leading to increased production of free radicals and neurotoxicity, while its overexpression inhibits brain inflammation^18^. In other studies, however, it has been shown to increase in cells with oxidative stress and promote death cell^4^. In addition, SIRT2 inhibition has been shown to protect against Parkinson’s disease and Huntington’s disease^19^,^20^. Previously published results have shown SIRT2 to be abundant in the brain and serum^9^,^11^,^21^,^22^ and our study provides further evidence for this and shows that it is also abundant in the guinea pig brain.

SIRT1 was the second-most abundant sirtuin in the brain and the only sirtuin detected in the CSF and plasma using our method. SIRT1 is a neuroprotective factor in a variety of models of neurodegenerative diseases, including Huntington’s disease, Multiple Sclerosis and AD^23^,^24^. It increases following exposure to cellular stressors, including energy/nutrient depletion and has been linked to increased lifespan in animal models^25^. We were only able to detect SIRT1 in both CSF and plasma while Kumar *et al*. have recently identified SIRT1, SIRT2 and SIRT3 in human serum using surface plasmon resonance^9^. The use of more sensitive mass spectrometry instrumentation, and/or fractionation or enrichment of plasma proteins, may improve sensitivity for the other sirtuins in plasma.

The mitochondrial sirtuins SIRT3-5 were found at lower levels in human brain but detection was improved after detergent fractionation. Further fractionation of samples or purification/enrichment of mitochondria may facilitate improved mitochondrial sirtuin quantification. Our data also show that SIRT6 and SIRT7 are the third and fourth most abundant members of the sirtuins family in the adult human frontal lobe. Both SIRT6 and SIRT7 have been associated with the maintenance of DNA stability and promotion of DNA repair following exposure to cellular stressors^26^,^27^.

We also confirm that mammalian sirtuins are localised to several subcellular compartments. While SIRT1, SIRT6 and SIRT7 are predominantly found in nuclear fractions, they are also detected in cytosol, cytoskeleton and membrane fractions, albeit at much lower levels. Similarly, although SIRT2 is the only human sirtuin primarily localised in the cytoplasm, it may also be found at lower levels in the nucleus and cellular membrane. Our data confirm that SIRT1 and SIRT2 may interact with both the nuclear and cytoplasmic subcellular compartments. Similarly, while the mitochondrial sirtuins (SIRT3-5) were previously reported to be exclusively localised to the mitochondria, other studies have reported the localisation of several variants in the nucleus as well as the cytoplasm, in line with our study^28^. SIRT5 has recently been found to have both desuccinylase and de-malonylase activities in both the mitochondria and cytosol^29^,^30^. The expression patterns of sirtuins in brain cells remains controversial and it is unclear whether certain sirtuins are specific to cell types. Our MRM study indicates that the majority of sirtuins (SIRT1, 2, 6 and 7) are present across all the main brain cell types.

For validation, analyses of the samples by immunohistochemical staining confirmed that all seven sirtuins are found in human primary astrocytes (Fig. 5, panel A row 1–5). PCR detected SIRT1 and SIRT2 mRNA in frontal, occipital and hippocampal brain tissue (Fig. 5, panel B). Western blotting identified protein levels in frontal lobe brain tissue with SIRT2 showing the strongest bands (Fig. 5, panel C and supplementary Figure S4), in good agreement with the results from our MRM assay (Fig. 3).

Various studies have demonstrated that abnormal changes to sirtuin proteins occur during disease states as well as ageing. SIRT2 accumulates with age in the mouse brain and spinal cord^22^. Other recent studies have shown that SIRT1 decreases with age in the rat brain^31^ and SIRT2 is upregulated in brain tumours^32^. Hence, there is great value in developing robust quantitative assays such as MRM mass spectrometry to accurately quantify these proteins in control vs disease states. Another important aspect of sirtuin biology is that their deacetylase activity is nicotinamide and NAD^+^ dependent, establishing a direct link between their function and energy metabolism. To improve sensitivity, we found our fractionation method of running the samples on SDS PAGE and excising gel bands corresponding to the molecular weights of the intact sirtuin proteins to improve detection sensitivity. The use of more sensitive mass spectrometers with high resolution MRM capability may improve detection of low abundant sirtuins and/or allow removal of the gel fractionation step. This would allow for sample lysates to be digested and analysed directly and decrease sample processing time. Using hybrid mass spectrometers capable of high resolution and accurate mass measurements such as the quadrupole-orbitrap may overcome these issues and yield better sensitivity for high-complexity samples. This will help future validation of levels in large cohorts which may be useful for the identification of sirtuins as potential biomarkers.

Collectively, our results add further insights to the limited data which currently exist regarding the expression of sirtuins in the human CNS. This technique provides a powerful tool and helps improve upon the limitations of current protocols. While mass spectrometry based assays for protein quantification may still have some barriers to overcome before they can be used in a clinical setting due to low throughput and expense compared to methods such as ELISA, they have great value in investigating lower abundance proteins such as sirtuins in complex samples such as the brain with great sensitivity and specificity. Furthermore the current approach could be extended to develop isotope specific sirtuin assays, or extended to other species by use of appropriate synthetic peptide standards. MRM mass spectrometry can also be utilised for the quantification of sirtuin related metabolites and thus targeted mass spectrometry methodologies have the potential to not only validate and complement currently established methods for investigation of sirtuin protein expression changes but can also help build a larger view of sirtuin biology in the normal brain and in disease conditions by linking sirtuin protein expression with metabolism.

## Methods

### Selection of target sirtuin peptides

Recombinant protein standards were purchased for seven human sirtuins (Cayman Chemical, USA), and 5 μg of each was run by 1D SDS-PAGE gel and colloidal coomassie stained. Sirtuin bands were excised, trypsin digested overnight followed by LC-MS/MS analysis on a QToF Ultima API hybrid tandem mass spectrometer (Micromass, UK) as previously described^33^,^34^,^35^ and a detailed method is provided in Supplementary Protocols. The two peptides with the highest signal intensity for each sirtuin were cross-referenced with Skyline software (MacCoss Lab Software, USA). Peptide sequences were checked to ensure no overlap with other sirtuins. Sirtuin standards were run using MRM LC-MS/MS on a 4000 Q TRAQ (SCIEX, USA) mass spectrometer to ensure good signals were detected for all 14 peptides selected for the final list (two unique peptides for each sirtuin). See Supplementary Tables S2 and S4 and supplementary protocols for peptide sequences and light and heavy product ions for all 14 peptides. The full list of transitions and corresponding collision energies and MRM method details are provided in Supplementary Table S5.

### Targeted mass spectrometry

MRM analyses were performed on a 4000 Q TRAP hybrid triple quadrupole linear ion trap mass spectrometer (SCIEX, USA) interfaced with a nanospray ion source, operating in positive ion mode and controlled by Analyst 1.5 software. Peptides were concentrated and desalted onto a micro C18 precolumn (500 μm × 2 mm, Michrom Bioresources, USA) with H_2_O:CH_3_CN (98:2, 0.05% TFA) at 15 μl/min. After a 4 min wash, the pre-column was automatically switched (10 port valve, Valco, USA) into line with a nano column (as described in the previous section). Peptides were eluted using a linear gradient of H_2_O:CH_3_CN (98:2, 0.1% formic acid) to H_2_O:CH_3_CN (36:64, 0.1% formic acid) at ~300 nl/min over 30 minutes. Samples were analyzed with an ion spray voltage of 2.4 kV, curtain gas flow of 12 and nebulizing gas flow of 5 L/min. Quadrupoles were operated in the low resolution mode, and the dwell time was 50 ms. For validation runs, the MRM experiment triggered MS/MS spectrum acquisition. MS/MS spectra were acquired in the trap mode (enhanced product ion) with dynamic fill time, Q1 was operated using low resolution. Each sirtuin protein (SIRT1-7, 2 peptides per protein) were run with 12 transition ions per run, per protein and with a 50 ms dwell time. See Supplementary Table S5 for a full list of transitions for each peptide and corresponding collision energies (estimated using Skyline software).

### Sirtuin Peptide Standards

Multiple point calibration was used where a series of standard ‘light’ peptides with known concentrations together with fixed amounts of stable isotope-labelled ‘heavy’ peptides (100 fmol/μl) were used to generate calibration curves (Fig. 1). The curves were expressed as ratios of light/heavy peak area (y-axis) versus concentration of light peptide (x-axis) for each of the 14 peptides selected. A commercially available stable isotope-labelled peptide standard (AQUA peptide, Sigma, USA) was used for absolute quantification of proteins. This heavy surrogate of each of the peptide standards is added at a constant level to all samples and standards and is used for correction of sample losses during workup and normalisation across runs, allowing accurate quantification of the target protein in samples. Peptides labelled with a stable isotope (^13^C and ^15^N) are chemically identical to their native counterparts and have identical chromatographic behaviour but can be distinguished from the calibration standards based on a small specific mass difference.

### Preparation of Cell Cultures, Tissues, CSF and Plasma

#### Cells

Adult human primary microglia, astrocytes, neurons and oligodendrocytes were cultured from resected normal adult brain tissue following removal of brain tumour with informed consent at the Minimally Invasive Cancer Centre, Prince of Wales Hospital, Sydney, Australia. Astrocytes were prepared from the mixed brain cell cultures using a protocol previously described by Guillemin *et al*.^36^. Cell culture procedures are described in detail in Supplementary Protocols. All cells and cell lines were lysed using RIPA buffer followed by probe sonication and cell debris removed by centrifugation at 10,000RPM for 5 min. Total protein concentration was assayed in the supernatant using the Pierce BCA protein assay kit (Life Technologies, Australia), and three 10 μg replicates were run by 1D SDS-PAGE followed by colloidal coomassie staining.

#### Human Tissues

20 μg of protein from five individual control post mortem brain tissue samples were extracted as described for cells in the previous section, and run on a 1D SDS-PAGE gel and coomassie stained. Detailed patient information including age, sex and post-mortem tissue collection times can be found in Supplementary Table S6. For the fractionated samples, 200 mg of frontal lobe brain tissue from five control subjects were pooled and differential detergent fractionation performed to obtain cytosol, nucleus, cytoskeletal and membrane protein fractions^37^. Each fraction (20 μg) was run on a 1D SDS-PAGE gel and coomassie stained.

#### Animals

Female guinea pigs (Dunkin–Hartley) and C57BL6 mice were housed in temperature-controlled rooms (21–22 °C; 49–55% humidity) with 12 h light-dark cycle (lights on 7:00–19:00). Food and water was available *ad libitum*. Wild type mice and guinea pig organs were lysed (RIPA buffer) by probe sonication. Proteins (20 μg/lane) were run by 1D SDS-PAGE and coomassie stained. Protein concentrations were determined using the Pierce BCA protein assay kit (Life Technologies, Australia).

#### CSF and plasma

CSF samples were collected by standard lumbar puncture from five patients assessed as clinically well after investigation for suspected meningitis (Supplementary Figure S5), returning normal results for routine CSF pathology markers (white cell count, protein, glucose and bacterial sterility). Aliquots (50 μl) from each patient were obtained for our study. Control human plasma from a healthy individual was immunodepleted of the six most abundant plasma proteins using an Hu6 column (Agilent, USA) according to manufacturer’s instructions^38^. For immunodepletion using the Multiple Affinity Removal System Hu6 column and buffer kit (Agilent, Santa Clara, CA, USA). 24 μl EDTA plasma was diluted into 120 μl Buffer A. 100 μl of this diluted EDTA plasma was injected onto the Hu6 column connected to a HP 1090 HPLC system (Agilent, Santa Clara, CA, USA) and the low abundance protein fraction was collected following manufacturer’s instructions. The low abundance protein fractions from six injections were pooled, buffer exchanged and concentrated into 45 mM NaHCO_3_ using Amicon 3 kDa centrifugal devices (Millipore, Billerica, MA, USA). CSF protein (5 μg) and depleted plasma protein (20 μg) as determined by a BCA protein assay were run on a 1D SDS-PAGE gel and colloidal coomassie stained.

For all samples, the band corresponding to the molecular weight for each sirtuin was excised (see Supplementary Figure S6), de-stained and trypsin digested overnight with heavy sirtuin peptides added as internal standards to all samples prior to digestion (the internal standard was added at a constant level to all samples and standards, and was at about the midrange of the standard curve). The tryptic peptides were dried under vacuum (Savant Speedvac, Thermo Scientific, USA), reconstituted in 0.1% formic acid (5 μl), injected into the mass spectrometer (1 μL) and analysed using MRM. Peak area ratios (light/heavy) were calculated for each endogenous peptide (light) relative to the spiked isotope-labelled (heavy) peptide using Skyline MRM analysis software. Protein concentrations were determined using calibration curves. All samples were fractionated by 1D-SDS-PAGE to reduce sample complexity. A workflow of the sample preparation procedure can be found in Supplementary Figure S3.

To test for matrix effects relevant to the in-gel processed sirtuins, Hu6 depleted plasma was run by SDS-PAGE (see Supplementary Figure S7) and sirtuin peptide standard curves prepared by spiking with excised gel bits taken from the expected migration position of the intact sirtuin. Additional matrices tested included buffer only (0.1% formic acid, no gel bit), a blank gel bit, and the spiked standard curves are shown in Supplementary Figure S2.

To determine recoveries of sirtuin proteins, commercial intact sirtuin standards were run by SDS PAGE (5 μg per lane). Hu6 high abundance depleted plasma spiked with 2 ug and 5 μg of the sirtuin standard for SIRT1, 2, 3, 5 and 6 were also run on the same gel (Supplementary Figure S8). Sirtuin 5 μg spike recoveries were calculated in the LAP spike relative to the sirtuin 5 μg standard protein in buffer only samples. The sirtuin 2 μg spike recoveries were calculated in the LAP spike relative to values extrapolated from the results of the sirtuin 5 μg standard protein in buffer only samples (Supplementary Table S1).

### Sirtuin mRNA Expression in Human Brain Tissue using PCR

For the gene expression studies RNA was extracted from human brain cells using RNeasy mini kits (Qiagen, Germany). The cDNA was prepared using SuperScript III First-Strand Synthesis System and random hexamers (Invitrogen Corporation, USA) as previously described^39^. Detailed protocol and primer sequences are described in Supplementary Protocols and Supplementary Table S7 respectively.

### Sirtuin Expression in Human Brain Cells and Tissue using immunohistochemical staining

Post mortem brain tissue from a male patient aged 63 years was obtained from the Sydney Brain Bank. Immunohistochemical staining was performed as previously published^40^ and was performed using anti-human sirtuin (1:250) primary antibodies (raised in rabbit). The full protocol description can be found in Supplementary Protocols.

### Sirtuin Protein Expression in human control brain tissue using western blotting

Protein from three individual control post mortem frontal brain tissue samples were extracted as described in the previous section, and run on a 1D SDS-PAGE gel (20 μg protein per lane), and western blotted with antibodies for SIRT1, SIRT2 and SIRT3 (details provided in Supplementary Protocols and list of antibodies used in Supplementary Table S8).

### Statistics

Sirtuin concentrations are presented as means ± SEM using peak area ratios of light and heavy peptides obtained from Skyline MRM Proteomics software v3.1 (MacCoss Lab, USA). Sirtuins were quantified based on an average of data from both peptides used for each sirtuin where possible. Statistical comparisons were performed using two-tailed student t-tests assuming equal variance. Differences between groups were considered statistically significant at the p < 0.05 level.

### Ethics

All human and animal brain tissue samples were obtained and experiments conducted in accordance with the guidelines of the National Health and Medical Research Council of Australia and were approved by the University of New South Wales Human Research Ethics Committee (human brain tissue reference number HC12563) and the University of New South Wales Animal Care Ethics Committee (guinea pig tissue reference number 14/40B and mice 13/39B). Adult human primary microglia, astrocytes, neurons and oligodendrocytes were cultured from resected normal adult brain tissue following removal of brain tumour with informed consent at the Minimally Invasive Cancer Centre, Prince of Wales Hospital, Sydney, Australia (reference number X12-0314 and HREC/12/RPAH/481). All control CSF samples were obtained with ethics approval from the Sydney Adventist Hospital, Sydney Australia, with informed consent obtained from all subjects (reference number SAHHREC #13-02).

## Additional Information

**How to cite this article**: Jayasena, T. *et al*. Application of Targeted Mass Spectrometry for the Quantification of Sirtuins in the Central Nervous System. *Sci. Rep.* **6**, 35391; doi: 10.1038/srep35391 (2016).

## Supplementary Material

Supplementary Information

## Figures and Tables

**Table 1:** Expression of sirtuins in the CNS and current methods used for analysis.

Name	Areas detected in CNS	Function in CNS	Techniques used
SIRT1	Human hippocampus and cortex^6^. Has also been detected in human serum at approx 8.16 ng/μl^8^. Mouse neural stem cells and adult mouse brain^7^. Porcine brain^21^.	Modulates memory formation and synaptic plasticity. Reduces with age in mice. Metabolic sensor.	TR-qPCR^6^, immunohistochemistry^41^, western blotting, surface plasmon resonance and ELISA^8^.
SIRT2	Mouse neural stem cells and adult mouse brain^7^. Porcine brain^21^. Human serum^9^.	Inhibitor of microglia-mediated inflammation and neurotoxicity^18^. Impairs neurite outgrowth and oligodendrocyte differentiation. Involved in myelin formation.	Mouse knockouts^18^, western blotting.
SIRT3	Cortex, Hippocampus and Cerebellum^10^. Mouse neural stem cells and adult mouse brain^7^. Porcine brain^21^. Rat brain. Human serum^9^.	Responses to oxidative stress and involved in maintenance of mitochondrial function.	Western blotting and qPCR^10^.
SIRT4	Mouse neural stem cells and adult mouse brain^7^. Porcine brain^21^. Rat cortical cells and brain tissue^42^.	Regulation of glial development^42^; involved in glutamate transport and protective role against excitotoxicity^43^.	qPCR, western blotting and immunofluorescence^21^,^42^
SIRT5	Mouse neural stem cells and adult mouse brain^7^. Porcine brain^21^.	SIRT5 gene polymorphism may promote molecular brain aging and be a risk factor for mitochondrial dysfunction-related diseases^44^.	qPCR^21^
SIRT6	Mainly localised in the nucleus in the cortical layers^45^. Mouse neural stem cells and adult mouse brain^7^,^46^,^47^. Rat brain. Porcine brain^21^.	Regulator of somatic growth by modulating neural chromatin and gene activity^46^. Modulated DNA repair in the brain^48^. Suppresses proinflammatory gene expression.	Brain specific mouse knockout models and primary brain cell models^46^,^48^,^49^; immunohistochemistry^45^; western blotting, immunofluorescence^47^.
SIRT7	Mouse neural stem cells and adult mouse brain^7^. Porcine brain^21^.	Positive regulator of RNA polymerase I transcription.	Mouse knockout^46^.
