# Supplementary material for: Application of Targeted Mass Spectrometry for the Quantification of Sirtuins in the Central Nervous System
Source: Sci Rep. 2016 Oct 20;6:35391. doi: 10.1038/srep35391 (PMC5071856; doi:10.1038/srep35391)

SUPPLEMENTARY INFORMATION

Application of Targeted Mass Spectrometry for the Quantification of Sirtuins in the Central Nervous System

T. Jayasena[a], A. Poljak[a,b,c], N. Braidy[a], L. Zhong[b], B. Rowlands[c], J. Muenchhoff[a], R. Grant[c,f], G. Smythe[c], C. Teo[d], M. Raftery[b], and P. Sachdev[a,e]*

[a]Centre for Healthy Brain Ageing (CHeBA), School of Psychiatry, University of New South Wales, Sydney, Australia

[b]Bioanalytical Mass Spectrometry Facility, Mark Wainwright Analytical Centre, University of New South Wales, Sydney, Australia

[c]School of Medical Sciences, University of New South Wales, Sydney, Australia

[d]Minimally Invasive Cancer Centre, Prince of Wales Hospital, Sydney, Australia

[e]Neuropsychiatric Institute, the Prince of Wales Hospital, Sydney, Australia

[f]Sydney Medical School, University of Sydney, Sydney, Australia

* Corresponding Author: Prof. P. Sachdev, Neuropsychiatric Institute, the Prince of Wales Hospital, Randwick NSW 2031, Australia. Tel: +61-2-93823763, Fax: +61-2-93823774

E-mail: p.sachdev@unsw.edu.au

SUPPLEMENTARY PROTOCOLS

a) Selection of Peptides

Digested peptides were separated by nano-LC using a Cap-LC autosampler system (Waters, USA). Samples (5 µl) were concentrated and desalted onto a micro C18 precolumn (500 µm x 2 mm, Michrom Bioresources, Auburn, CA) with H_2O:CH_3CN (98:2, 0.05% HFBA) at 15 ul/min. After a 4 min wash, the pre-column was automatically switched (Valco 10 port valve, Houston, TX) into line with a fritless nano column (75 µm x ~12 cm) containing Magic C18 (~10cm, 200Å, Michrom). Peptides were eluted using a linear gradient of H_2O:CH_3CN (98:2, 0.1% formic acid) to H_2O:CH_3CN (55:45, 0.1% formic acid) at ~300 nl/min over 30 min. The precolumn was connected via a fused silica capillary (10 cm, 25 µ) to a low volume tee (Upchurch Scientific) where high voltage (2400 V) was applied and the column tip positioned ~1 cm from the Z-spray inlet of an QTof Ultima API hybrid tandem mass spectrometer (Micromass, UK). Positive ions were generated by electrospray and the QTof operated in data dependent acquisition mode (DDA). A Tof MS survey scan was acquired (m/z 350-1700, 1 s) and the two largest multiply charged ions (counts > 20) were sequentially selected by Q1 for MS-MS analysis. Argon was used as collision gas and an optimum collision energy chosen (based on charge state and mass). Tandem mass spectra were accumulated for up to 2 s (m/z 50-2000). Peak lists were submitted to the database search program Mascot (Matrix Science, UK) to confirm identification of sirtuin peptides.

b) Sirtuin standards and calibration curves

Isotopically labelled internal standards are excellent tools to normalise the variability in sample handling, injection volume and the performance of the mass spectrometer. Synthetic light and heavy peptides were purchased from GL Biochem (China) and Sigma-Aldrich (USA) respectively. Calibration curves in the range of 1-200 fmol/µl (three replicates using three transitions) were prepared. Heavy peptide (100 fmol/µl) was added to all standards and peptide ratios (light/heavy) were obtained using Skyline MRM analysis software. Average inter- and intra- assay %CV values (n=3) were calculated for each of the seven sirtuins. The linear range, limit of detection (LOD) and limit of quantification (LOQ) were determined. A signal-to-noise ratio of >3:1 and >10:1 was used to define LOD and LOQ respectively.

Cell Culture

Cells were cultured in medium RPMI 1640 supplemented with 10% foetal bovine serum, 1% 1-glutamax, 1% antibacterial/antifungal, and 0.5% glucose. Cells were maintained at 37°C in a

humidified atmosphere containing 95% air/5% CO_2. Cells were seeded into 24-well tissue culture plates to a density of 1×10^5 cells 24 hours prior to experimentation. Neurons were prepared from the same mixed brain cell cultures as previously described (Guillemin et al., 2007). Briefly, cells were plated in 24-well culture plates coated with Matrigel (1/20 in Neurobasal) and maintained in Neurobasal medium supplemented with 1% B-27 supplement, 1% Glutamax, 1% antibiotic/antifungal, 0.5% HEPES buffer, and 0.5% glucose. Microglia were prepared from the mixed brain cell cultures using a published protocol (Guillemin et al., 2001). Briefly, the original mixed brain cell culture was shaken at 220 rpm for 2 h, floating cells were centrifuged, transferred to Permanox chamber slides (NUNC) at a density of 5×10^5 cells/ml and grown in DMEM supplemented with 10% fetal calf serum (FCS), 1% L-glutamax, 0.5% glucose, IL-3 (300 IU/ml), macrophage colony-stimulating factor (M-CSF) (50 IU/ml), and 1% antibiotic/antifungal. Oligodendrocytes were prepared from the mixed brain cell cultures using a published protocol (Guillemin et al., 2001). Briefly, cells were seeded and grown in flasks coated with poly-L-lysine to confluence at 34°C, the permissive temperature, in DMEM/F12 supplemented with 10% FBS, 1% antibiotic/antifungal, 1% of G418 (Invitrogen, Melbourne, Australia) and then shifted to 39°C, the non-permissive temperature that leads to the 'differentiated' state for 72 hours. Cells were cultured in medium RPMI supplemented with 1% myelin binding protein, 1% 1-glutamax, and 1% antibacterial/antifungal. CNS cell lines of neurons (SKNSH), astrocytes (U251), oligodendrocytes (M310) and microglia (CHM5) were cultured in medium RPMI supplemented with 10% foetal bovine serum, 1% 1-glutamax, 1% antibacterial/antifungal, and 0.5% glucose. All cells were maintained at 37°C in a humidified atmosphere containing 95% air/5% CO_2. Cells were seeded into 24-well tissue culture plates (3 wells per cell type) to a density of 1×10^5 cells 24 hours prior to experimentation. Wells were pooled prior to cell lysis and probe sonication and all measurements were performed in triplicate.

PCR

Briefly, for each reaction 2 µl of diluted cDNA, 10 µL of SYBR green master mix, 0.15 µL of 10 µM forward and reverse primers and 7.7 µl of nuclease-free water was used making a total volume of 20 µl. Q-PCR was carried out using the Mx3500P Real-Time PCR system (Stratagene, Australia). The primer sequences are shown in Supplementary Table S7. The relative expression levels of sirtuin transcripts were calculated using a mathematical model based on the individual Q-PCR primer efficiencies and the quantified values were normalized against the housekeeping gene 18S. From

these values, fold-differences in the levels of transcripts between individual cell cultures were calculated according to the formula $2^{-\Delta\Delta Ct}$

Immunohistochemical Staining

Formalin fixed blocks from the hippocampal region were embedded in paraffin and cut into 5 µm sections on superfrost plus slides. For sirtuin staining, sections were deparaffinised in xylene for 20 min and rehydrated through graded alcohol treatment. Antigen retrieval was performed through the use of Target Retrieval solution pH 6 (DAKO, Denmark) and autoclaving at 121°C for 20 min. Endogenous peroxidase was blocked using 3% w/v hydrogen peroxide for 5 min and endogenous biotin was blocked as described previously. After incubation with 10% horse serum for 30 min to block non-specific binding, sections were incubated with anti-human sirtuin (1:250) primary antibodies (raised in rabbit), for 2 hours at ambient temperature. After three rinses, sections were incubated with secondary antibodies–biotinylated goat-anti rabbit or mouse (Vector Laboratories, USA, 1:200) for 30 min at ambient temperature followed by 30 min treatment with avidin-biotin-complex (ABC) elite (Vector). Labelling was visualized with liquid DAB and sections were counterstained and mounted. Negative controls for non-specific staining included replacement of the primary antibody by normal rabbit or mouse IgG.

Western Blotting

The gel was electroblotted (20V, 110mA, 90mins) onto a nitrocellulose membrane in transfer buffer of 50mM tris, 40mM glycine, 1.3mM SDS, 20% methanol, pH 9.2. After transfer of proteins, the membrane was incubated in 10% skim milk powder solution containing primary sirtuin antibody. SIRT1, SIRT2 and SIRT3 rabbit polyclonal antibodies were used (1:1000 dilution, Abcam, UK), followed by a secondary antibody (1:100,000 dilution of anti-mouse IgG, Pierce, USA). See Supplementary Table S8 for full antibody details and dilutions. Chemiluminescence blots were developed using 1:1 solution of Super Signal West Femto luminol and peroxide buffer (Pierce, USA) according to manufacturer's instructions.

References

Guillemin, G.J. *et al* Characterization of the kynurenine pathway in human neurons. *J Neurosci* **27**, 12884-92 (2007).

Guillemin, G. J. *et al.* Kynurenine pathway metabolism in human astrocytes: a paradox for neuronal protection. *J Neurochem* **78**, 1-13 (2001).

Supplementary Table S1. Sirtuin recovery using the full protocol (sirtuins run by SDS/PAGE) with spiking into matrices consisting of either buffer only (5μg sirtuin) or low abundance plasma (LAP) proteins with either 5μg or 2μg sirtuin spiked. Sirtuin 5μg spike recoveries were calculated in the LAP spike relative to sirtuin 5μg standard protein in buffer only samples. Sirtuin 2μg spike recoveries were calculated in the LAP spike relative to values extrapolated from the results of the sirtuin 5μg standard protein in buffer only samples. The peak ratios and percentage recovery values is from three transitions used for each of the two sirtuin peptides per sirtuin protein and were obtained from one gel lane per sirtuin protein. The gel on which these samples were run is shown in Figure S8.

	**Avg Peak Intensity Ratio (Light/Heavy)**	**Avg Peak Intensity Ratio (Light/Heavy)**	**% Recovery**	**%Recovery**
	**DINTIEDAVK**	**TSVAGTVR**	**DINTIEDAVK**	**TSVAGTVR**
SIRT1 Std 5μg	0.0106	0.0196		
SIRT1 Std 2μg (extrapolated)	0.0042	0.0078		
SIRT1 Std 5μg + 20μg LAP	0.0080	0.0178	76.10	90.79
SIRT1 Std 2μg + 20μg LAP	0.0046	0.0101	108.50	128.83
	**LLDELTLEGVAR**	**IFSEVTPK**	**LLDELTLEGVAR**	**IFSEVTPK**
SIRT2 Std 5μg	0.0656	0.0514		
SIRT2 Std 2μg (extrapolated)	0.0262	0.0206		
SIRT2 Std 5μg + 20μg LAP	0.0523	0.0496	79.70	96.34
SIRT2 Std 2μg + 20μg LAP	0.0245	0.0219	93.51	106.31
	**LYTQNIDGLER**	**VSGIPASK**	**LYTQNIDGLER**	**VSGIPASK**
SIRT3 Std 5μg	0.1015	0.0085		
SIRT3 Std 2μg (extrapolated)	0.0406	0.0034		
SIRT3 Std 5μg + 20μg LAP	0.0620	0.0067	61.08	78.80
SIRT3 Std 2μg + 20μg LAP	0.0236	0.0035	58.13	102.9
	**VVVITQNIDELHR**	**NLLEIHGSLFK**	**VVVITQNIDELHR**	**NLLEIHGSLFK**
SIRT5 Std 5μg	0.0224	0.0271		
SIRT5 Std 2μg (extrapolated)	0.00896	0.01084		
SIRT5 Std 5μg + 20μg LAP	0.01812	0.0198	80.89	73.33
SIRT5 Std 2μg + 20μg LAP	0.0093	0.0104	103.80	95.94

	FLVSQNVDGLHVR	LVIVNLQPTK	FLVSQNVDGLHVR	LVIVNLQPTK
SIRT6 Std 5μg	0.0789	0.0837		
SIRT6 Std 2μg (extrapolated)	0.0316	0.0335		
SIRT6 Std 5μg + 20μg LAP	0.0571	0.0743	72.37	88.76
SIRT6 Std 2μg + 20μg LAP	0.0326	0.0459	103.16	131.14

Supplementary Table S2. Human sirtuin peptide homology with guinea pig and mouse. Sequence variations from the human are highlighted in red.

Sirtuin	Human Peptide	Homology in Guinea Pig and Mice
**SIRT1**	DINTIEDAVK	Identical sequence found in both Guinea Pig and Mouse
	TSVAGTVR	In Mice: TSVAETVR<br>In GP: TSVAETVR
**SIRT2**	LLDELTLEGVAR	Identical sequence found in Guinea PigIn Mice: LLDELTLEGVTR
	IFSEVTPK	In mice: IFSEATPR<br>In GP: LFSDVTPK
**SIRT3**	LYTQNIDGLER	Identical sequence found in both Guinea Pig and Mouse
	VSGIPASK	In mice: ASGIPASK<br>In GP: ASGIPASK
**SIRT4**	RPIQHGDFVR	In mice: RPIQHIDFVR<br>In GP: RPIQHSDFVR
	FILTAWEK	In mice: FILTAREQ<br>In GP: FTLTAQDK
**SIRT5**	VVVITQNIDELHR	Identical sequence found in Mouse<br>In GP: VAVITQNIDELHLR
	NLLEIHGSLFK	In mice: NLLEIHGTLFK<br>In GP: NLVEIHGTIFK
**SIRT6**	FLVSQNVDGLHVR	Identical sequence found in both Guinea Pig and Mouse
	LVIVNLQPTK	Identical sequence found in both Guinea Pig and Mouse
**SIRT7**	LLAESADLVTELQGR	In mice: LLAESEDLVTELQGR<br>In GP: LLAESEDLVTELQGR
	DTIVHFGER	Identical sequence found in both Guinea Pig and Mouse

Supplementary Table S3. Sequences of all 7 human sirtuins and their isoforms (obtained from UniProt). The peptides used for MRM quantification experiments are highlighted in red and in larger font size. All sirtuin isoforms contain at least one of the peptides used for quantification, and both peptides are represented in the majority of isoforms.

**SIRT1 Isoform 1**	>sp|Q96EB6|SIR1_HUMAN NAD-dependent protein deacetylase sirtuin-1 OS=Homo sapiens GN=SIRT1 PE=1 SV=2<br>MADEAALALQPGGSPSAAGADREAASSPAGEPLRKRPRRDGPGLERSPGEPGGAAPER<br>EVPAAARGCPGAAAAALWREAEAEAAAAGGEQEAQATAAAGEGDNGPGLQGPSREP<br>PLADNLYDEDDDDEGEEEEEAAAAAIGYRDNLLFGDEIITNGFHSCESDEEDRASHASSS<br>DWTPRPRIGPYTFVQQHLMIGTDPRTILKDLLPETIPPPELDDMTLWQIVINILSEPPKRK<br>KRK**DINTIEDAVK**LLQECKKIIVLTGAGVSVSCGIPDFRSRDGIYARLAVDFPDLPDPQA<br>MFDIEYFRKDPRPFFKFAKEIYPGQFQPSLCHKFIALSDKEGKLLRNYTQNIDTLEQVAGIQ<br>RIIQCHGSFATASCLICKYKVDCEAVRGDIFNQVVPRCPRCPADEPLAIMKPEIVFFGENL<br>PEQFHRAMKYDKDEVDLLIVIGSSLKVRPVALIPSSIPHEVPQILINREPLPHLHFDVELLGD<br>CDVIINELCHRLGGEYAKLCCNPVKLSEITEKPPRTQKELAYLSELPPTPLHVSEDSSSPERT<br>SPPDSSVIVTLLDQAAKSNDDLDVSESKGCMEEKPQEVQTSRNVESIAEQMENPDLKNV<br>GSSTGEKNER**TSVAGTVR**KCWPNRVAKEQISRRLDGNQYLFLPPNRYIFHGAEVYSDS<br>EDDVLSSSSCGSNSDSGTCQSPSLEEPMEDESEIEEFYNGLEDEPDVPERAGGAGFGTDG<br>DDQEAINEAISVKQEVTDMNYPSNKS
**SIRT1 Isoform 2**	>sp|Q96EB6-2|SIR1_HUMAN Isoform 2 of NAD-dependent protein deacetylase sirtuin-1 OS=Homo sapiens GN=SIRT1<br>MADEAALALQPGGSPSAAGADREAASSPAGEPLRKRPRRDGPGLERSPGEPGGAAPER<br>EVPAAARGCPGAAAAALWREAEAEAAAAGGEQEAQATAAAGEGDNGPGLQGPSREP<br>PLADNLYDEDDDDEGEEEEEAAAAAIGYRDNLLFGDEIITNGFHSCESDEEDRASHASSS<br>DWTPRPRIGPYTFVQQHLMIGTDPRTILKDLLPETIPPPELDDMTLWQIVINILSEPPKRK<br>KRK**DINTIEDAVK**LLQECKKIIVLTGAGVSVSCGIPDFRSRDGIYARLAVDFPDLPDPQA<br>MFDIEYFRKDPRPFFKFAKEIYPGQFQPSLCHKFIALSDKEGKLLRNYTQNIDTLEQVAGIQ<br>RIIQCHGSFATASCLICKYKVDCEAVRGDIFNQVVPRCPRCPADEPLAIMKPEIVFFGENL<br>PEQFHRAMKYDKDEVDLLIVIGSSLKVRPVALIPSNQYLFLPPNRYIFHGAEVYSDSEDDV<br>LSSSSCGSNSDSGTCQSPSLEEPMEDESEIEEFYNGLEDEPDVPERAGGAGFGTDGDDQ<br>EAINEAISVKQEVTDMNYPSNKS
**SIRT2 Isoform 1**	>sp|Q8IXJ6|SIR2_HUMAN NAD-dependent protein deacetylase sirtuin-2 OS=Homo sapiens GN=SIRT2 PE=1 SV=2<br>MAEPDPSHPLETQAGKVQEAQDSDSDSEGGAAGGEADMDFLRNLFSQTLSLGSQKER<br>**LLDELTLEGVAR**YMQSERCRRVICLVGAGISTSAGIPDFRSPSTGLYDNLEKYHLPYPE<br>AIFEISYFKKHPEPFFALAKELYPGQFKPTICHYFMRLLKDKGLLLRCYTQNIDTLERIAGLE<br>QEDLVEAHGTFYTSHCVSASCRHEYPLSWMKEK**IFSEVTPK**CEDCQSLVKPDIVFFGES<br>LPARFFSCMQSDFLKVDLLLVMGTSLQVQPFASLISKAPLSTPRLLINKEKAGQSDPFLG<br>MIMGLGGGMDFDSKKAYRDVAWLGECDQGCLALAELLGWKKELEDLVRREHASIDAQ<br>SGAGVPNPSTSASPKKSPPPAKDEARTTEREKPQ

**SIRT2 Isoform 2**	>sp|Q8IXJ6-2|SIR2_HUMAN Isoform 2 of NAD-dependent protein deacetylase sirtuin-2 OS=Homo sapiens GN=SIRT2<br>MDFLRNLFSQTLSLGSQKER**LLDELTLEGVAR**YMQSERCRRVICLVGAGISTSAGIPDF<br>RSPSTGLYDNLEKYHLPYPEAIFEISYFKKHPEPFFALAKELYPGQFKPTICHYFMRLLKDKG<br>LLLRCYTQNIDTLERIAGLEQEDLVEAHGTFYTSHCVSASCRHEYPLSWMKEK**IFSEVTP**<br>**K**CEDCQSLVKPDIVFFGESLPARFFSCMQSDFLKVDLLLVMGTSLQVQPFASLISKAPLST<br>PRLLINKEKAGQSDPFLGMIMGLGGGMDFDSKKAYRDVAWLGECDQGCLALAELLGW<br>KKELEDLVRREHASIDAQSGAGVPNPSTSASPKKSPPPAKDEARTTEREKPQ
**SIRT2 Isoform 3**	>sp|Q8IXJ6-3|SIR2_HUMAN Isoform 3 of NAD-dependent protein deacetylase sirtuin-2 OS=Homo sapiens GN=SIRT2<br>MPLAECPSCRCLSSFRSVDFLRNLFSQTLSLGSQKER**LLDELTLEGVAR**YMQSERCRR<br>VICLVGAGISTSAGIPDFRSPSTGLYDNLEKYHLPYPEAIFEISYFKKHPEPFFALAKELYPGQ<br>FKPTICHYFMRLLKDKGLLLRCYTQNIDTLERIAGLEQEDLVEAHGTFYTSHCVSASCRHE<br>YPLSWMKEK**IFSEVTPK**CEDCQSLVKPDIVFFGESLPARFFSCMQSDFLKVDLLLVMGT<br>SLQVQPFASLISKAPLSTPRLLINKEKAGQSDPFLGMIMGLGGGMDFDSKKAYRDVAWL<br>GECDQGCLALAELLGWKKELEDLVRREHASIDAQSGAGVPNPSTSASPKKSPPPAKDEA<br>RTTEREKPQ
**SIRT2 Isoform 4**	>sp|Q8IXJ6-4|SIR2_HUMAN Isoform 4 of NAD-dependent protein deacetylase sirtuin-2 OS=Homo sapiens GN=SIRT2<br>MAEPDPSHPLETQAGKVQEAQDSDSDSEGGAAGGEADMDFLRNLFSQTLSLGSQKER<br>**LLDELTLEGVAR**YMQSERCRRVICLVGAGISTSAGIPDFRSPSTGLYDNLEKYHLPYPE<br>AIFEISYFKKHPEPFFALAKELYPGQFKPTICHYFMRLLKDKGLLLRCYTQNIDTLERIAGLE<br>QEDLVEAHGTFYTSHCVSASCRHEYPLSWMKEK**IFSEVTPK**CEDCQSLVKPDIVFFGES<br>LPARFFSCMQSDFLKVDLLLVMGTSLQGRGLAG
**SIRT2 Isoform 5**	>sp|Q8IXJ6-5|SIR2_HUMAN Isoform 5 of NAD-dependent protein deacetylase sirtuin-2 OS=Homo sapiens GN=SIRT2<br>MAEPDRRRVICLVGAGISTSAGIPDFRSPSTGLYDNLEKYHLPYPEAIFEISYFKKHPEPFFA<br>LAKELYPGQFKPTICHYFMRLLKDKGLLLRCYTQNIDTLERIAGLEQEDLVEAHGTFYTSHC<br>VSASCRHEYPLSWMKEK**IFSEVTPK**CEDCQSLVKPDIVFFGESLPARFFSCMQSDFLKV<br>DLLLVMGTSLQVQPFASLISKAPLSTPRLLINKEKAGQSDPFLGMIMGLGGGMDFDSKK<br>AYRDVAWLGECDQGCLALAELLGWKKELEDLVRREHASIDAQSGAGVPNPSTSASPKK<br>SPPPAKDEARTTEREKPQ
**SIRT3 Isoform 1**	>sp|Q9NTG7|SIR3_HUMAN NAD-dependent protein deacetylase sirtuin-3, mitochondrial OS=Homo sapiens GN=SIRT3 PE=1 SV=2<br>MAFWGWRAAAALRLWGRVVERVEAGGGVGPFQACGCRLVLGGRDDVSAGLRGSHG<br>ARGEP<br>LDPARPLQRPPRPEVPRAFRRQPRAAAPSFFFSSIKGGRRSISFSVGASSVVGSGGSSDKG<br>KLSLQDVAELIRARACQRVVVMVGAGISTPSGIPDFRSPGSGLYSNLQQYDLPYPEAIFEL<br>PFFFHNPKPFFTLAKELYPGNYKPNVTHYFLRLLHDKGLLLR**LYTQNIDGLERVSGIPA**<br>**SK**LVEAHGTFASATCTVCQRPFPGEDIRADVMADRVPRCPVCTGVVKPDIVFFGEPLPQ<br>RFLLHVVDFPMADLLLILGTSLEVEPFASLTEAVRSSVPRLLINRDLVGPLAWHPRSRDVA

	QLGDVVHGVESLVELLGWTEEMRDLVQRETGKLDGPDK
**SIRT3 Isoform 2**	>sp|Q9NTG7-2|SIR3_HUMAN Isoform 2 of NAD-dependent protein deacetylase sirtuin-3, mitochondrial OS=Homo sapiens GN=SIRT3<br>MVGAGISTPSGIPDFRSPGSGLYSNLQQYDLPYPEAIFELPFFFHNPKPFFTLAKELYPGNY<br>KPNVTHYFLRLLHDKGLLLR**LYTQNIDGLERVSGIPASK**LVEAHGTFASATCTVCQR<br>PFPGEDIRADVMADRVPRCPVCTGVVKPDIVFFGEPLPQRFLLHVVDFPMADLLLILGTS<br>LEVEPFASLTEAVRSSVPRLLINRDLVGPLAWHPRSRDVAQLGDVVHGVESLVELLGWT<br>EEMRDLVQRETGKLDGPDK
**SIRT4**	>sp|Q9Y6E7|SIR4_HUMAN NAD-dependent protein lipoamidase sirtuin-4, mitochondrial OS=Homo sapiens GN=SIRT4 PE=1 SV=1<br>MKMSFALTFRSAKGRWIANPSQPCSKASIGLFVPASPPLDPEKVKELQRFITLSKRLLVM<br>TGAGISTESGIPDYRSEKVGLYARTDR**RPIQHGDFVR**SAPIRQRYWARNFVGWPQFS<br>SHQPNPAHWALSTWEKLGKLYWLVTQNVDALHTKAGSRRLTELHGCMDRVLCLDCGE<br>QTPRGVLQERFQVLNPTWSAEAHGLAPDGDVFLSEEQVRSFQVPTCVQCGGHLKPDV<br>VFFGDTVNPDKVDFVHKRVKEADSLLVVGSSLQVYSGYR**FILTAWEK**KLPIAILNIGPTR<br>SDDLACLKLNSRCGELLPLIDPC
**SIRT5 Isoform 1**	>sp|Q9NXA8|SIR5_HUMAN NAD-dependent protein deacylase sirtuin-5, mitochondrial OS=Homo sapiens GN=SIRT5 PE=1 SV=2<br>MRPLQIVPSRLISQLYCGLKPPASTRNQICLKMARPSSSMADFRKFFAKAKHIVIISGAGV<br>SAESGVPTFRGAGGYWRKWQAQDLATPLAFAHNPSRVWEFYHYRREVMGSKEPNAG<br>HRAIAECETRLGKQGRR**VVVITQNIDELHR**KAGTK**NLLEIHGSLFK**TRCTSCGVVA<br>ENYKSPICPALSGKGAPEPGTQDASIPVEKLPRCEEAGCGGLLRPHVVWFGENLDPAILE<br>EVDRELAHCDLCLVVGTSSVVYPAAMFAPQVAARGVPVAEFNTETTPATNRFRFHFQG<br>PCGTTLPEALACHENETVS
**SIRT5 Isoform 2**	>sp|Q9NXA8-2|SIR5_HUMAN Isoform 2 of NAD-dependent protein deacylase sirtuin-5, mitochondrial OS=Homo sapiens GN=SIRT5<br>MRPLQIVPSRLISQLYCGLKPPASTRNQICLKMARPSSSMADFRKFFAKAKHIVIISGAGV<br>SAESGVPTFRGAGGYWRKWQAQDLATPLAFAHNPSRVWEFYHYRREVMGSKEPNAG<br>HRAIAECETRLGKQGRR**VVVITQNIDELHR**KAGTK**NLLEIHGSLFK**TRCTSCGVVA<br>ENYKSPICPALSGKGAPEPGTQDASIPVEKLPRCEEAGCGGLLRPHVVWFGENLDPAILE<br>EVDRELAHCDLCLVVGTSSVVYPAAMFAPQVAARGVPVAEFNTETTPATNRFSHLISISS<br>LIIIKN
**SIRT5 Isoform 3**	>sp|Q9NXA8-3|SIR5_HUMAN Isoform 3 of NAD-dependent protein deacylase sirtuin-5, mitochondrial OS=Homo sapiens GN=SIRT5<br>MRPLQIVPSRLISQLYCGLKPPASTRNQICLKMARPSSSMADFRKFFAKAKHIVIISGAGV<br>SAESGVPTFRGAGGYWRKWQAQDLATPLAFAHNPSRVWEFYHYRREVMGSKEPNAG<br>HRAIAECETRLGKQGRR**VVVITQNIDELHR**KAGTK**NLLEIHGSLFK**TRCTSCGVVA<br>ENYKSPICPALSGKGCEEAGCGGLLRPHVVWFGENLDPAILEEVDRELAHCDLCLVVGTS<br>SVVYPAAMFAPQVAARGVPVAEFNTETTPATNRFRFHFQGPCGTTLPEALACHENETV<br>S
**SIRT5 Isoform 4**	>sp|Q9NXA8-4|SIR5_HUMAN Isoform 4 of NAD-dependent protein deacylase

	sirtuin-5, mitochondrial OS=Homo sapiens GN=SIRT5<br>MGSKEPNAGHRAIAECETRLGKQGRR**VVVITQNIDELHR**KAGTK**NLLEIHGSLFK**T<br>RCTSCGVVAENYKSPICPALSGKGAPEPGTQDASIPVEKLPRCEEAGCGGLLRPHVVWF<br>GENLDPAILEEVDRELAHCDLCLVVGTSSVVYPAAMFAPQVAARGVPVAEFNTETTPAT<br>NRFRFHFQGPCGTTLPEALACHENETVS
**SIRT6 Isoform 1**	>sp|Q8N6T7|SIR6_HUMAN NAD-dependent protein deacetylase sirtuin-6<br>OS=Homo sapiens GN=SIRT6 PE=1 SV=2<br>MSVNYAAGLSPYADKGKCGLPEIFDPPEELERKVWELARLVWQSSSVVFHTGAGISTAS<br>GIPDFRGPHGVWTMEERGLAPKFDTTFESARPTQTHMALVQLERVGLLR**FLVSQNV**<br>**DGLHVR**SGFPRDKLAELHGNMFVEECAKCKTQYVRDTVVGTMGLKATGRLCTVAKA<br>RGLRACRGELRDTILDWEDSLPDRDLALADEASRNADLSITLGTSLQIRPSGNLPLATKRR<br>GGR**LVIVNLQPTK**HDRHADLRIHGYVDEVMTRLMKHLGLEIPAWDGPRVLERALPPL<br>PRPPTPKLEPKEESPTRINGSIPAGPKQEPCAQHNGSEPASPKRERPTSPAPHRPPKRVKA<br>KAVPS
**SIRT6 Isoform 2**	>sp|Q8N6T7-2|SIR6_HUMAN Isoform 2 of NAD-dependent protein<br>deacetylase sirtuin-6 OS=Homo sapiens GN=SIRT6<br>MSVNYAAGLSPYADKGKCGLPEIFDPPEELERKVWELARLVWQSSSVVFHTGAGISTAS<br>GIPDFRGPHGVWTMEERGLAPKFDTTFESARPTQTHMALVQLERVGLLR**FLVSQNV**<br>**DGLHVR**SGFPRDKLAELHGNMFVEECAKCKTQYVRDTVVGTMGLKATGRLCTVAKA<br>RGLRACRNADLSITLGTSLQIRPSGNLPLATKRRGGR**LVIVNLQPTK**HDRHADLRIHGY<br>VDEVMTRLMKHLGLEIPAWDGPRVLERALPPLPRPPTPKLEPKEESPTRINGSIPAGPKQ<br>EPCAQHNGSEPASPKRERPTSPAPHRPPKRVKAKAVPS
**SIRT7 Isoform 1**	>sp|Q9NRC8|SIR7_HUMAN NAD-dependent protein deacetylase sirtuin-7<br>OS=Homo sapiens GN=SIRT7 PE=1 SV=1<br>MAAGGLSRSERKAAERVRRLREEQQRERLRQVSRILRKAAAERSAEEGR**LLAESADLV**<br>**TELQGR**SRRREGLKRRQEEVCDDPEELRGKVRELASAVRNAKYLVVYTGAGISTAASIP<br>DYRGPNGVWTLLQKGRSVSAADLSEAEPTLTHMSITRLHEQKLVQHVVSQNCDGLHLR<br>SGLPRTAISELHGNMYIEVCTSCVPNREYVRVFDVTERTALHRHQTGRTCHKCGTQLR**D**<br>**TIVHFGER**GTLGQPLNWEAATEAASRADTILCLGSSLKVLKKYPRLWCMTKPPSRRPKL<br>YIVNLQWTPKDDWAALKLHGKCDDVMRLLMAELGLEIPAYSRWQDPIFSLATPLRAGE<br>EGSHSRKSLCRSREEAPPGDRGAPLSSAPILGGWFGRGCTKRTKRKKVT
**SIRT7 Isoform 2**	>sp|Q9NRC8-2|SIR7_HUMAN Isoform 2 of NAD-dependent protein<br>deacetylase sirtuin-7 OS=Homo sapiens GN=SIRT7<br>MAAGGLSRSERKAAERVRRLREEQQRERLRQVSRILRKAAAERSAEEGR**LLAESADLV**<br>**TELQGR**SRRREGLKRRQEEVCDDPEELRGKVRELASAVRNAKYLVVYTGAGISTAASIP<br>DYRGPNGVWTLLQKGRSVSAADLSEAEPTLTHMSITRLHEQKLVRALGGWYTCQGPGR<br>APWCPVGN
**SIRT7 Isoform 3**	>sp|Q9NRC8-3|SIR7_HUMAN Isoform 3 of NAD-dependent protein<br>deacetylase sirtuin-7 OS=Homo sapiens GN=SIRT7<br>MPGPRRRSPSACPQVSRILRKAAAERSAEEGR**LLAESADLVTELQGR**SRRREGLKRR

	QEEVCDDPEELRGKVRELASAVRNAKYLVVYTGAGISTAASIPDYRGPNGVWTLLQKGR<br>SVSAADLSEAEPTLTHMSITRLHEQKLVQHVVSQNCDGLHLRSGLPRTAISELHGNMYIE<br>VCTSCVPNREYVRVFDVTERTALHRHQTGRTCHKCGTQLRDTIVHFGERGTLGQPLNW<br>EAATEAASRADTILCLGSSLKVLKKYPRLWCMTKPPSRRPKLYIVNLQWTPKDDWAALKL<br>HGKCDDVMRLLMAELGLEIPAYSRVL

Supplementary Table S4. List of 14 unique sirtuin peptides selected for sirtuin protein quantification. Arg and Lys amino acids highlighted in bold used in ‘heavy’ peptides, labelled with a stable isotope (^{13}C and ^{15}N). Precursor charge state for all peptides was 2^+.

Sirtuin	Peptide	Light Precursor Ion *m/z*	Heavy Precursor Ion *m/z*
SIRT1	DINTIEDAVK	559.2904	563.2975
	TSVAGTVR	395.7245	400.7286
SIRT2	LLDEELTLEGVAR	664.8746	669.8788
	IFSEVTPK	460.758	464.7651
SIRT3	LYTQNIDGLER	661.341	666.3451
	VSGIPASK	379.724	383.7311
SIRT4	RPIQHGDFVR	612.8334	617.8376
	FILTAWEK	504.2817	508.2888
SIRT5	VVVITQNIDELHR	512.6229	515.9589
	NLLEIHGSLFK	635.8613	639.8613
SIRT6	FLVSQNVDGLHVR	742.4044	747.4086
	LVIVNLQPTK	562.8555	566.8626
SIRT7	LLAESADLVTELQGR	807.9385	812.9426
	DTIVHFGER	537.2724	542.2765

Supplementary Table S5. List of product and transition ions with collision energies used for all 14 selected sirtuin peptides. Each sirtuin protein (SIRT1-7, with 2 peptides per protein) was run with 12 transition ions per run, per protein and with a 50ms dwell time. Precursor charge state for all peptides was 2^+.

Precursor Ion *m/z*	Transition Ion	Peptide	Declustering Potential	Collision Energy
559.2904	889.4625	SIRT1.DINTIEDAVK.y8.light	71.9	27.6
559.2904	775.4196	SIRT1.DINTIEDAVK.y7.light	71.9	27.6
559.2904	674.3719	SIRT1.DINTIEDAVK.y6.light	71.9	27.6
563.2975	897.4767	SIRT1.DINTIEDAVK.y8.heavy	71.9	27.6
563.2975	783.4338	SIRT1.DINTIEDAVK.y7.heavy	71.9	27.6
563.2975	682.3861	SIRT1.DINTIEDAVK.y6.heavy	71.9	27.6
395.7245	602.362	SIRT1.TSVAGTVR.y6.light	60	18.3
395.7245	503.2936	SIRT1.TSVAGTVR.y5.light	60	18.3
395.7245	432.2565	SIRT1.TSVAGTVR.y4.light	60	18.3
400.7286	612.3703	SIRT1.TSVAGTVR.y6.heavy	60	18.3
400.7286	513.3019	SIRT1.TSVAGTVR.y5.heavy	60	18.3
400.7286	442.2648	SIRT1.TSVAGTVR.y4.heavy	60	18.3
664.8746	858.5043	SIRT2.LLDELTLEGVAR.y8.light	79.6	33.6
664.8746	745.4203	SIRT2.LLDELTLEGVAR.y7.light	79.6	33.6
664.8746	644.3726	SIRT2.LLDELTLEGVAR.y6.light	79.6	33.6
669.8788	868.5126	SIRT2.LLDELTLEGVAR.y8.heavy	79.6	33.6
669.8788	755.4285	SIRT2.LLDELTLEGVAR.y7.heavy	79.6	33.6
669.8788	654.3809	SIRT2.LLDELTLEGVAR.y6.heavy	79.6	33.6
460.758	807.4247	SIRT2.IFSEVTPK.y7.light	64.7	22
460.758	660.3563	SIRT2.IFSEVTPK.y6.light	64.7	22
460.758	573.3243	SIRT2.IFSEVTPK.y5.light	64.7	22
464.7651	815.4389	SIRT2.IFSEVTPK.y7.heavy	64.7	22
464.7651	668.3705	SIRT2.IFSEVTPK.y6.heavy	64.7	22
464.7651	581.3385	SIRT2.IFSEVTPK.y5.heavy	64.7	22
661.341	944.4796	SIRT3.LYTQNIDGLER.y8.light	79.3	33.4
661.341	816.421	SIRT3.LYTQNIDGLER.y7.light	79.3	33.4
661.341	702.3781	SIRT3.LYTQNIDGLER.y6.light	79.3	33.4
666.3451	954.4879	SIRT3.LYTQNIDGLER.y8.heavy	79.3	33.4
666.3451	826.4293	SIRT3.LYTQNIDGLER.y7.heavy	79.3	33.4
666.3451	712.3863	SIRT3.LYTQNIDGLER.y6.heavy	79.3	33.4
379.724	659.3723	SIRT3.VSGIPASK.y7.light	58.8	17.4
379.724	572.3402	SIRT3.VSGIPASK.y6.light	58.8	17.4
379.724	515.3188	SIRT3.VSGIPASK.y5.light	58.8	17.4
383.7311	667.3865	SIRT3.VSGIPASK.y7.heavy	58.8	17.4

383.7311	580.3544	SIRT3.VSGIPASK.y6.heavy	58.8	17.4
383.7311	523.333	SIRT3.VSGIPASK.y5.heavy	58.8	17.4
612.8334	1068.559	SIRT4.RPIQHGDFVR.y9.light	75.8	30.7
612.8334	971.5057	SIRT4.RPIQHGDFVR.y8.light	75.8	30.7
612.8334	858.4217	SIRT4.RPIQHGDFVR.y7.light	75.8	30.7
617.8376	1078.567	SIRT4.RPIQHGDFVR.y9.heavy	75.8	30.7
617.8376	981.514	SIRT4.RPIQHGDFVR.y8.heavy	75.8	30.7
617.8376	868.4299	SIRT4.RPIQHGDFVR.y7.heavy	75.8	30.7
504.2817	747.4036	SIRT4.FILTAWEK.y6.light	67.9	24.5
504.2817	634.3195	SIRT4.FILTAWEK.y5.light	67.9	24.5
504.2817	533.2718	SIRT4.FILTAWEK.y4.light	67.9	24.5
508.2888	755.4178	SIRT4.FILTAWEK.y6.heavy	67.9	24.5
508.2888	642.3337	SIRT4.FILTAWEK.y5.heavy	67.9	24.5
508.2888	541.286	SIRT4.FILTAWEK.y4.heavy	67.9	24.5
512.6229	896.4585	SIRT5.VVVITQNIDELHR.y7.light	68.5	23
512.6229	782.4155	SIRT5.VVVITQNIDELHR.y6.light	68.5	23
512.6229	669.3315	SIRT5.VVVITQNIDELHR.y5.light	68.5	23
515.9589	906.4667	SIRT5.VVVITQNIDELHR.y7.heavy	68.5	23
515.9589	792.4238	SIRT5.VVVITQNIDELHR.y6.heavy	68.5	23
515.9589	679.3397	SIRT5.VVVITQNIDELHR.y5.heavy	68.5	23
635.8613	930.5043	SIRT5.NLLEIHGSLFK.y8.light	77.5	32
635.8613	801.4618	SIRT5.NLLEIHGSLFK.y7.light	77.5	32
635.8613	688.3777	SIRT5.NLLEIHGSLFK.y6.light	77.5	32
639.8684	938.5185	SIRT5.NLLEIHGSLFK.y8.heavy	77.5	32
639.8684	809.4759	SIRT5.NLLEIHGSLFK.y7.heavy	77.5	32
639.8684	696.3919	SIRT5.NLLEIHGSLFK.y6.heavy	77.5	32
742.4044	1124.581	SIRT6.FLVSQNVDGLHVR.y10.light	85.2	38.1
742.4044	909.4901	SIRT6.FLVSQNVDGLHVR.y8.light	85.2	38.1
742.4044	696.3787	SIRT6.FLVSQNVDGLHVR.y6.light	85.2	38.1
747.4086	1134.589	SIRT6.FLVSQNVDGLHVR.y10.heavy	85.2	38.1
747.4086	919.4984	SIRT6.FLVSQNVDGLHVR.y8.heavy	85.2	38.1
747.4086	706.387	SIRT6.FLVSQNVDGLHVR.y6.heavy	85.2	38.1
562.8555	912.5513	SIRT6.LVIVNLQPTK.y8.light	72.1	27.8
562.8555	799.4672	SIRT6.LVIVNLQPTK.y7.light	72.1	27.8
562.8555	700.3988	SIRT6.LVIVNLQPTK.y6.light	72.1	27.8
566.8626	920.5655	SIRT6.LVIVNLQPTK.y8.heavy	72.1	27.8
566.8626	807.4814	SIRT6.LVIVNLQPTK.y7.heavy	72.1	27.8
566.8626	708.413	SIRT6.LVIVNLQPTK.y6.heavy	72.1	27.8
807.9385	915.5258	SIRT7.LLAESADLVTELQGR.y8.light	90	41.8
807.9385	802.4417	SIRT7.LLAESADLVTELQGR.y7.light	90	41.8
807.9385	703.3733	SIRT7.LLAESADLVTELQGR.y6.light	90	41.8

812.9426	925.5341	SIRT7.LLAESADLVTELQGR.y8.heavy	90	41.8
812.9426	812.45	SIRT7.LLAESADLVTELQGR.y7.heavy	90	41.8
812.9426	713.3816	SIRT7.LLAESADLVTELQGR.y6.heavy	90	41.8
537.2724	958.5105	SIRT7.DTIVHFGER.y8.light	70.3	26.4
537.2724	857.4628	SIRT7.DTIVHFGER.y7.light	70.3	26.4
537.2724	744.3787	SIRT7.DTIVHFGER.y6.light	70.3	26.4
542.2765	968.5188	SIRT7.DTIVHFGER.y8.heavy	70.3	26.4
542.2765	867.4711	SIRT7.DTIVHFGER.y7.heavy	70.3	26.4
542.2765	754.387	SIRT7.DTIVHFGER.y6.heavy	70.3	26.4

Supplementary Table S6. Human frontal lobe brain tissue sample and subject details.

Patient	Sex	Age at death	Post mortem tissue collection time
1	Male	72	24h
2	Female	71	24h
3	Male	72	24h
4	Male	71	29h
5	Male	54	24h

Supplementary Table S7. Real-time primer sequences used for PCR analysis.

Primer name	Oligo name	Sequence (5'→3')
SIRT1-Forw	NM_012238-Forw	CAC-CAG-AAA-GAA-CTT-CAC-CAC-CAG
SIRT1-Rev	NM_012238-Rev	ACC-ATC-AAG-CCG-CCT-ACT-AAT-CTG
SIRT2-Forw	AJ505014-Forw	AGG-GAC-AAG-GAG-CAG-GGT-TC
SIRT2-Rev	AJ505014-Rev	GAA-GAG-AGA-CAG-CGG-CAG-GAC
SIRT3-Forw	NM_12239-Forw	GAG-GTT-CTT-GCT-GCA-TGT-GGT-TG
SIRT3-Rev	NM_12239-Rev	AGT-TTC-CCG-CTG-CAC-AAG-GTC
SIRT4-Forw	NM_012240-Forw	TTG-TGC-CAG-CAA-GTC-CTC-CTC
SIRT4-Rev	NM_012240-Rev	GTC-TCT-TGG-AAA-GGG-TGA-TGA-AGC
SIRT5-Forw	NM_12241-Forw	TCC-AGC-GTC-CAC-ACG-AAA-CC
SIRT5-Rev	NM_12241-Rev	AAC-ACC-AGC-TCC-TGA-GAT-GAT-GAC
SIRT6-Forw	NM_016539-Forw	GCT-GGA-GCC-CAA-GGA-GGA-ATC
SIRT6-Rev	NM_016539-Forw	AGT-AAC-AAA-GTG-AGA-CCA-CGA-GAG
SIRT7-Forw	NM_016538-Forw	GAG-CCA-ACC-CTC-ACC-CAC-ATG
SIRT7-Rev	NM_016538-Rev	ACG-CAG-GAG-GTA-CAG-ACT-TCA-ATG
GAPDH-Forw	NM_017008-Forw	TGG-AGT-CTA-CTG-GCG-TCT-T
GAPDH –Rev	NM_017008-Rev	TGT-CAT-ATT-TCT-CGT-GGT-TCA

Supplementary Table S8. Antibodies and dilutions used for western blotting.

Antibody	Dilution	Manufacturer
Rabbit Polyclonal SIRT1	1:1000	Abcam (Cambridge, UK)
Rabbit Polyclonal SIRT2	1:1000	Abcam (Cambridge, UK)
Rabbit Polyclonal SIRT3	1:1000	Abcam (Cambridge, UK)

Supplementary Figure S1. Representative chromatograms for all 14 sirtuin peptides used in the MRM method, showing transition ions, retention times and signal intensities. The vertical dotted lines either side of the peak clusters indicate the boundaries for peak area integration.

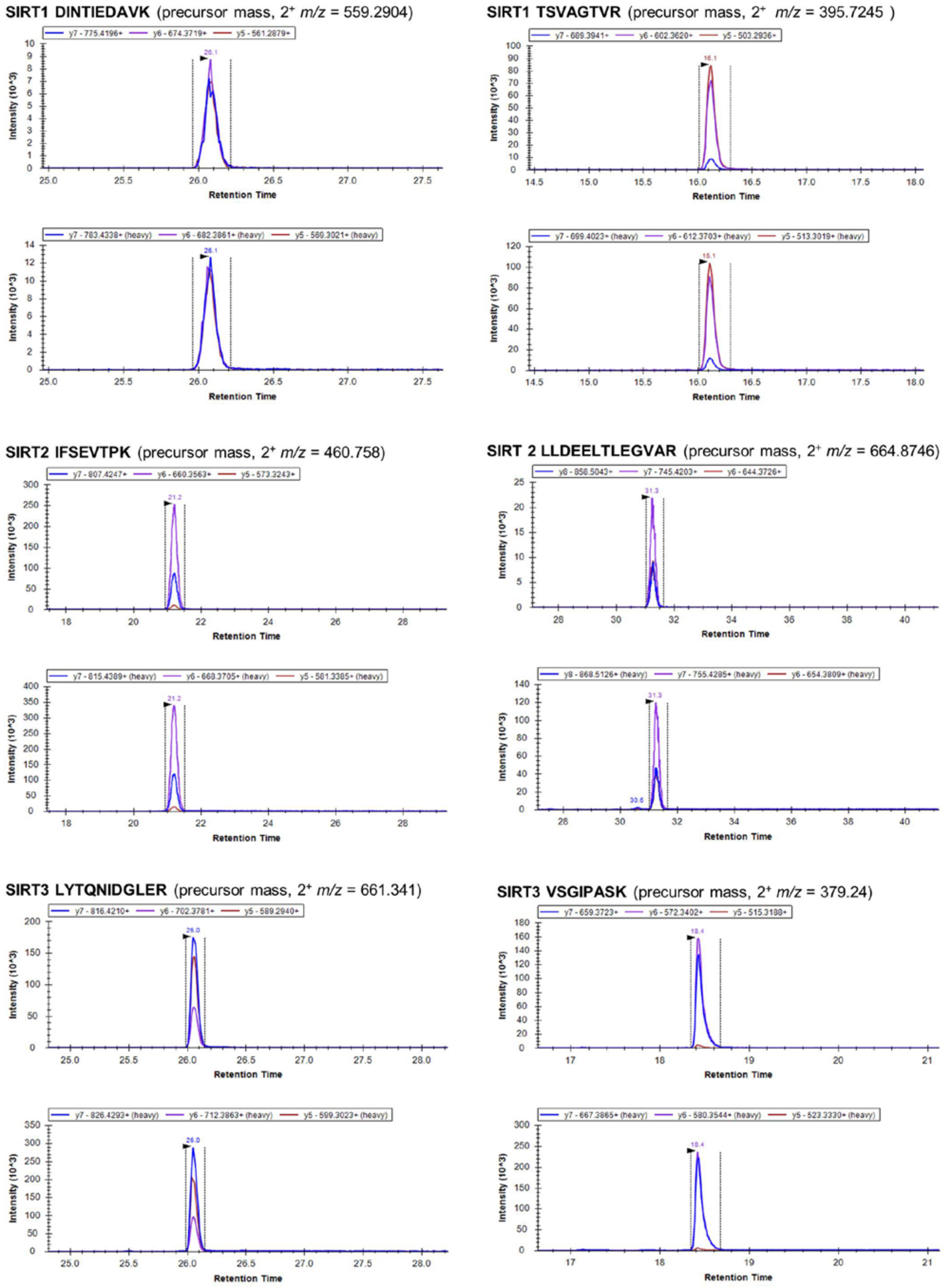

Supplementary Figure S1 continued.

SIRT4 FILTAWEK (precursor mass, 2^+ m/z = 612.8334)

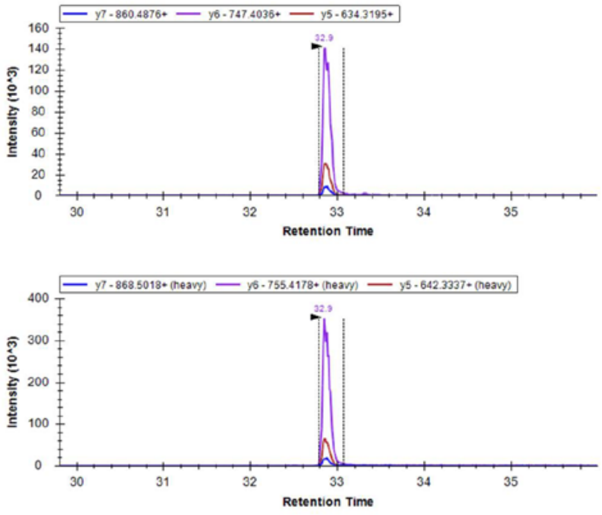

SIRT5 VVVITQNIDELHR (precursor mass, 2^+ m/z = 512.6229)

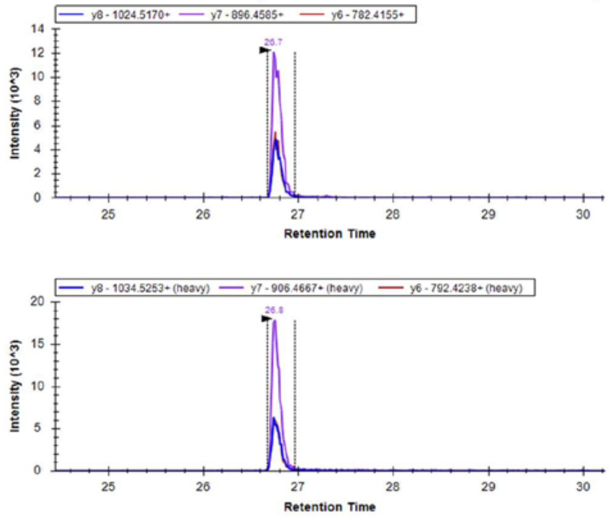

SIRT5 NLLEIHGSLFK (precursor mass, 2^+ m/z = 635.8613)

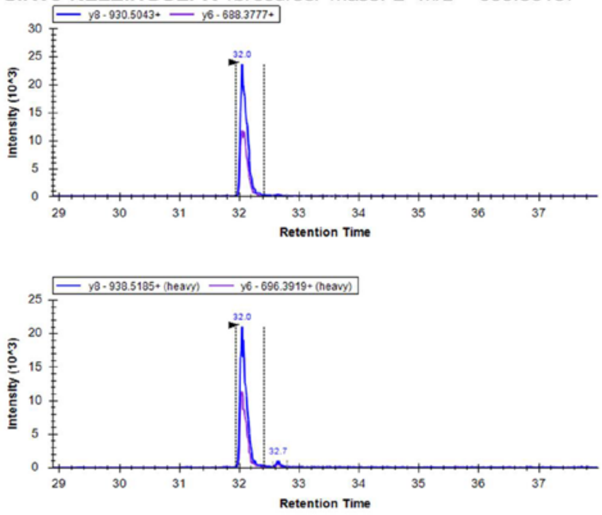

SIRT6 FLVSQNVDGLHVR (precursor mass, 2^+ m/z = 742.4044)

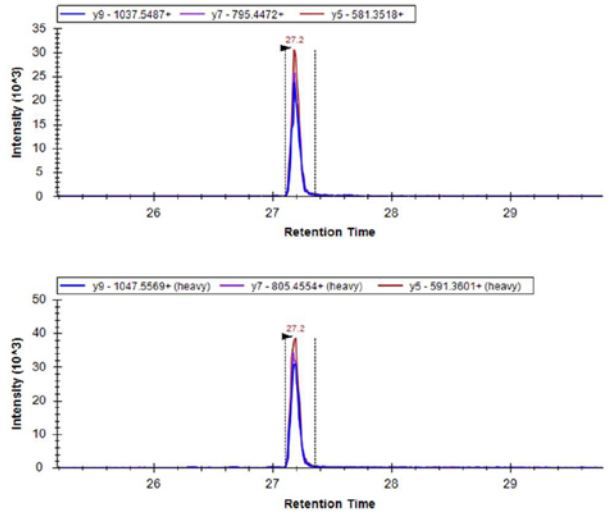

SIRT6 LVIVNLQPTK (precursor mass, 2^+ m/z = 562.8555)

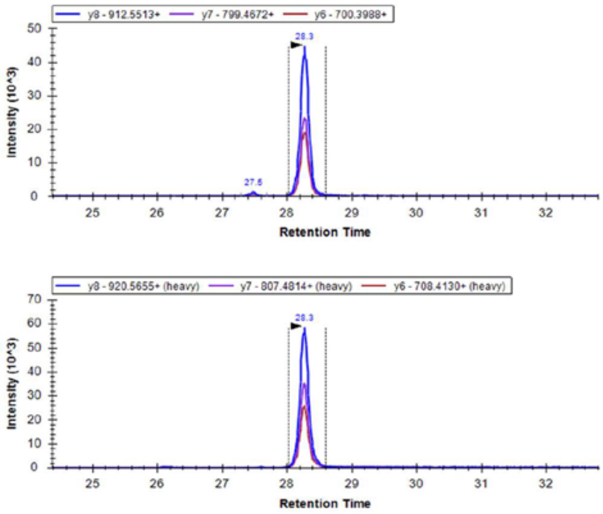

Supplementary Figure S1 continued.

SIRT7 DTIVHFGER (precursor mass, 2^+ m/z = 537.2724)

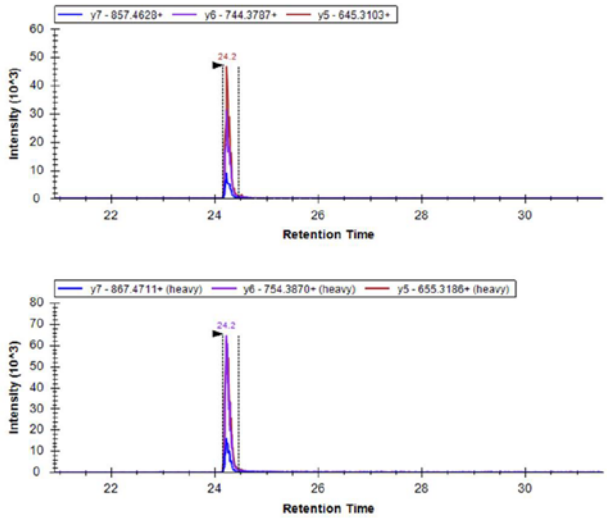

SIRT7 LLAESADLVTELQGR (precursor mass, 2^+ m/z = 807.9385)

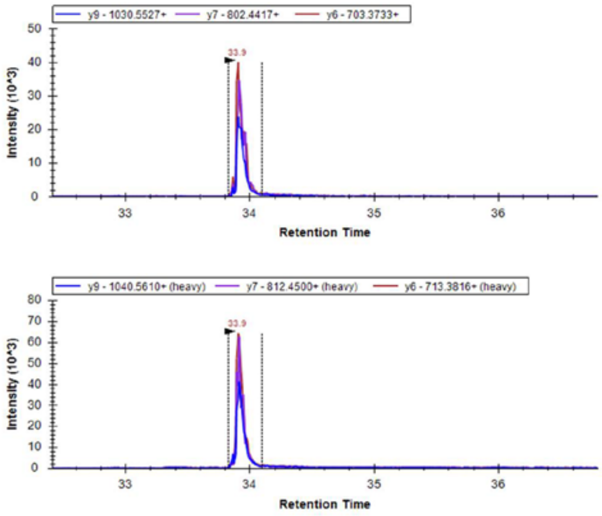

Supplementary Figure S2. Sirtuin peptide standard curves prepared in several matrices, including buffer only, a blank gel control, a gel containing low abundance plasma proteins (see Supplementary Figure S7 for the gel used for the gel matrices).

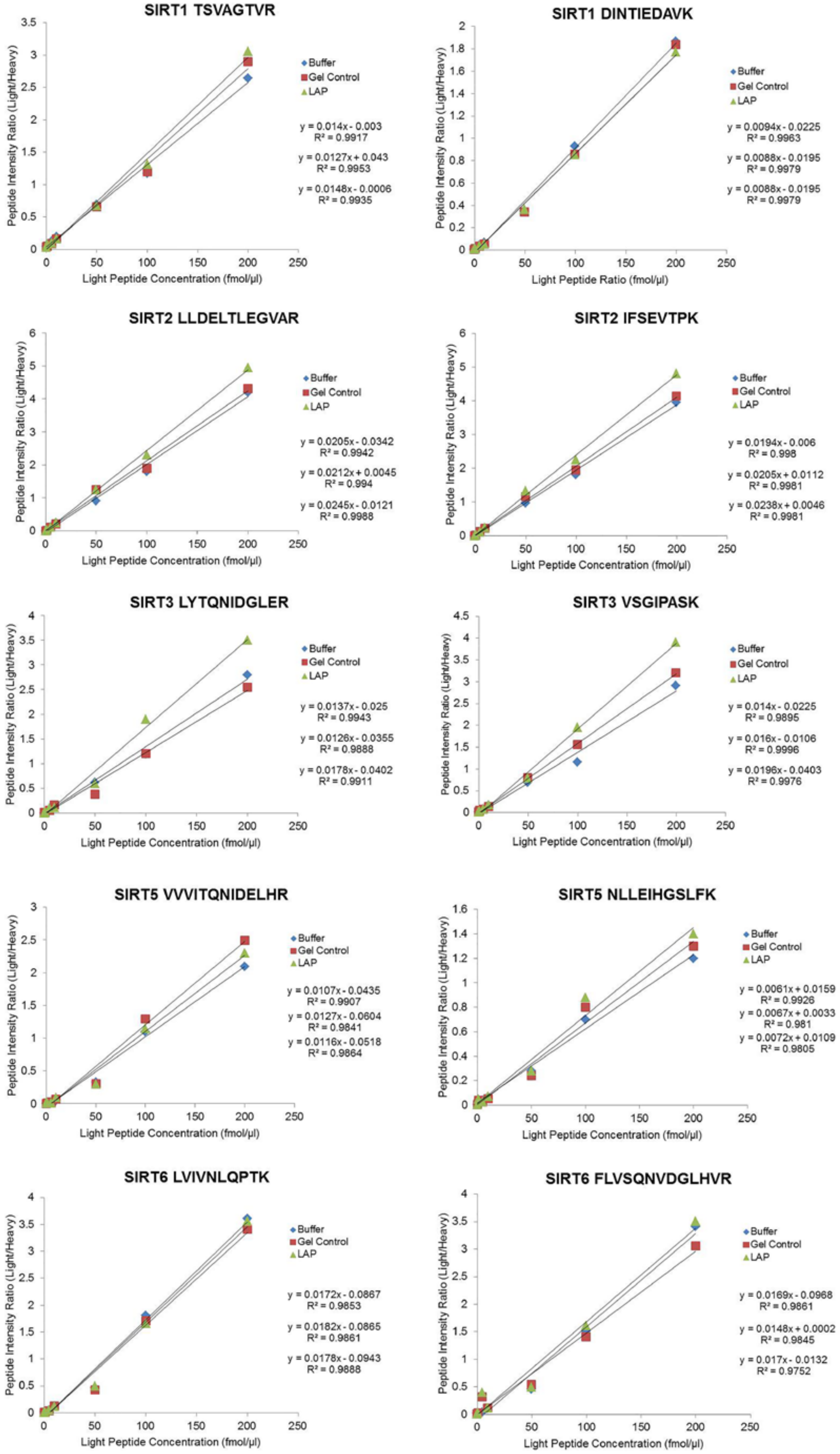

Supplementary Figure S3. Sample fractionation and MRM workflow.

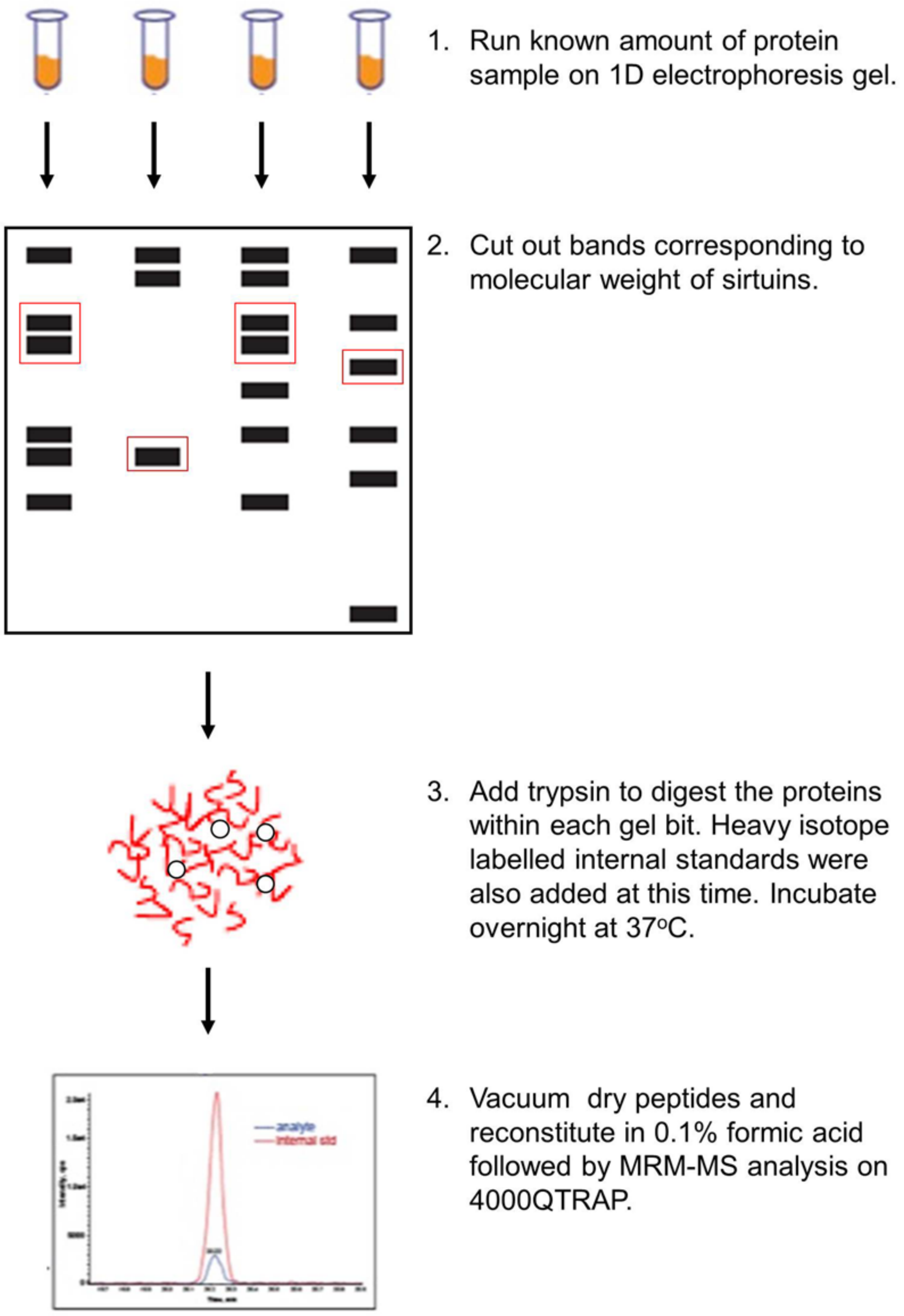

Supplementary Figure S4. Full length western blots of SIRT1-3 protein expression in human control frontal lobe brain tissue (n=3 individuals) at molecular weights of approx 40kDa, 50kDa and 30kDa respectively. All blots were run under the same experimental protocols with 1:1000 dilution of primary antibodies. The exposure times shown are 3hrs, 30sec and overnight for SIRT1, SIRT2 and SIRT3 respectively.

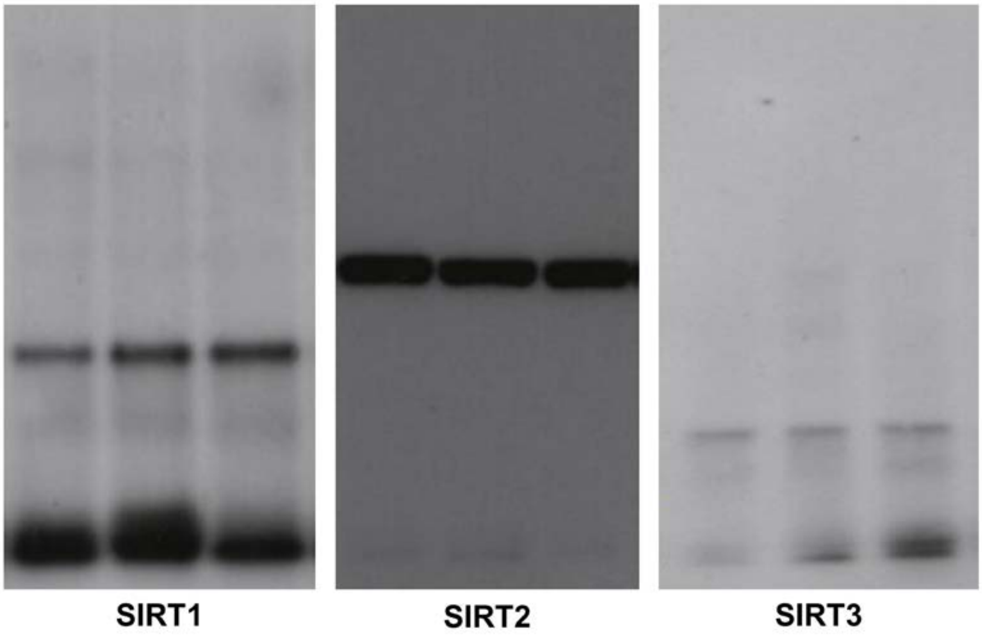

Supplementary Figure S5. SIRT1 levels in CSF and depleted plasma. SIRT1 levels in CSF (n=5) samples and a plasma sample depleted of the six most abundant plasma proteins (high abundance protein removal was achieved using an Agilent Hu6 affinity column).

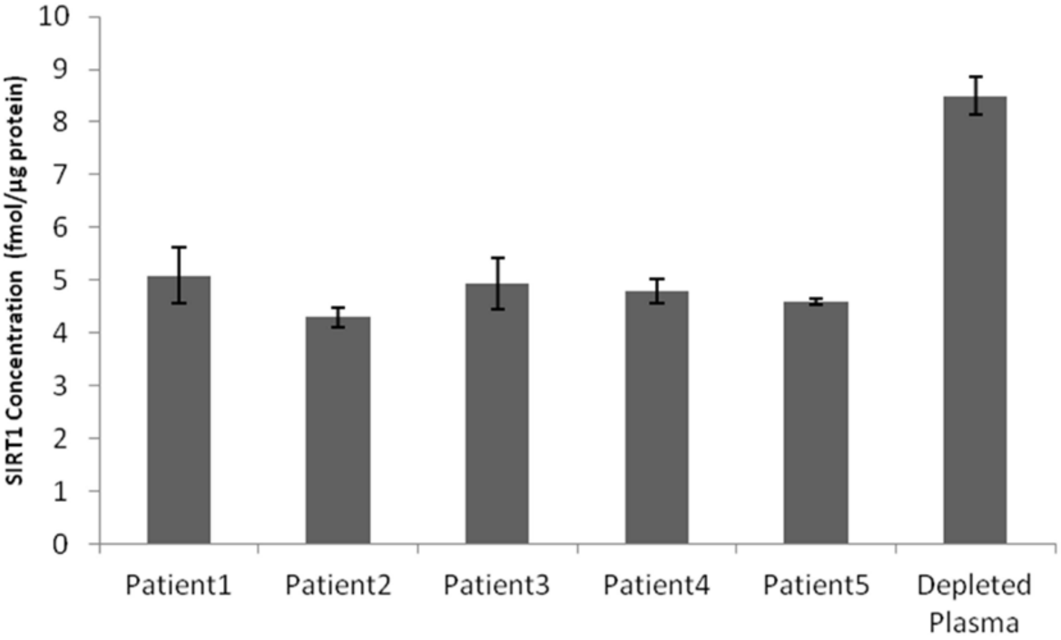

Supplementary Figure S6. Representative colloidal coomassie stained SDS PAGE gel of recombinant sirtuin protein standards highlighted in red boxes with the bands cut corresponding to the molecular weight of each full length sirtuin. Note the presence of host cell contaminants in most of the preparations, especially SIRT7, and the particularly low yield of SIRT4. These preparations were adequate to validate good signal intensity for each of the two peptides per sirtuin used for MRM quantification. SIRT1, 2, 3, 5 and 6 were also used to estimate recoveries using the full protocol (see Supplementary Figure S7 and Supplementary Table S1), in buffer only *vs* LAP spiked samples.

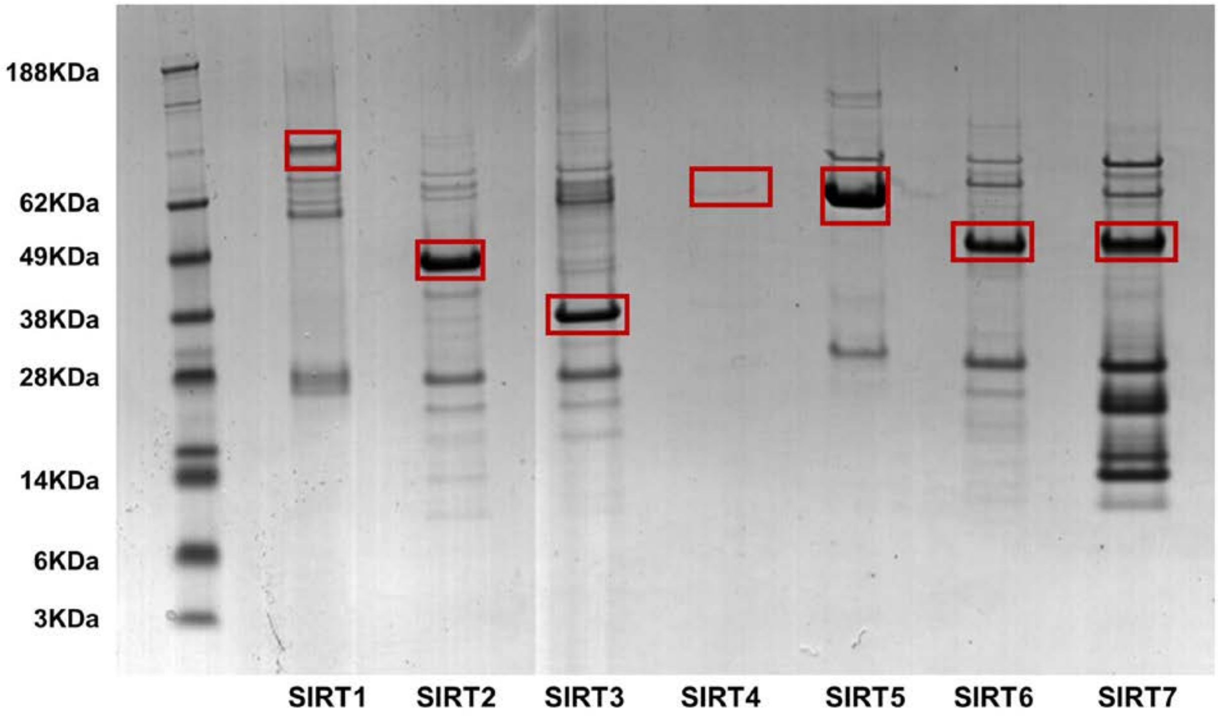

Supplementary Figure S7. A typical colloidal coomassie stained SDS/PAGE gel used to assess matrix effects on standard curves. Bands were cut from positions at which intact sirtuin proteins would typically be expected, and used to test matrix effects on the synthetic peptide standards. In addition to this, intact commercial recombinant sirtuin preparations were spiked into depleted plasma and run by SDS/PAGE (see Supplementary Figure S8).

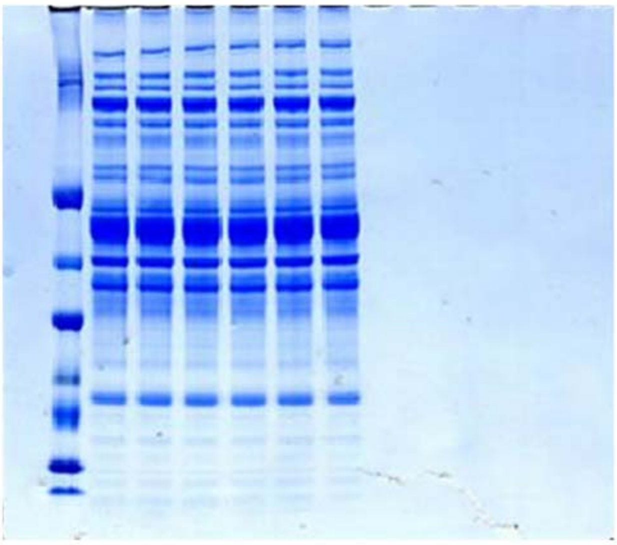

Supplemententay Figure S8. Colloidal oomassie stained SDS/PAGE gel used for sirtuin recovery experiments. Lane 1: prestained molecular weight markers; Lanes 2-6: commercial SIRT1, 2, 3, 5 and 6 recombinant sirtuin protein standards (5ug per lane. This amount was based on the manufacturer's indicated quantification of vial contents.); Lanes 8-12: 20ug Depleted plasma spiked with 5ug of sirtuin standards 1, 2, 3, 5 and 6; Lanes 14-18: Depleted plasma spiked with 2ug of sirtuin standards 1, 2, 3, 5 and 6. Sirtuins 4 and 7 were not used for this experiment as the SIRT4 yield was very low, and SIRT7 was particularly impure (see Supplementary Figure S6).

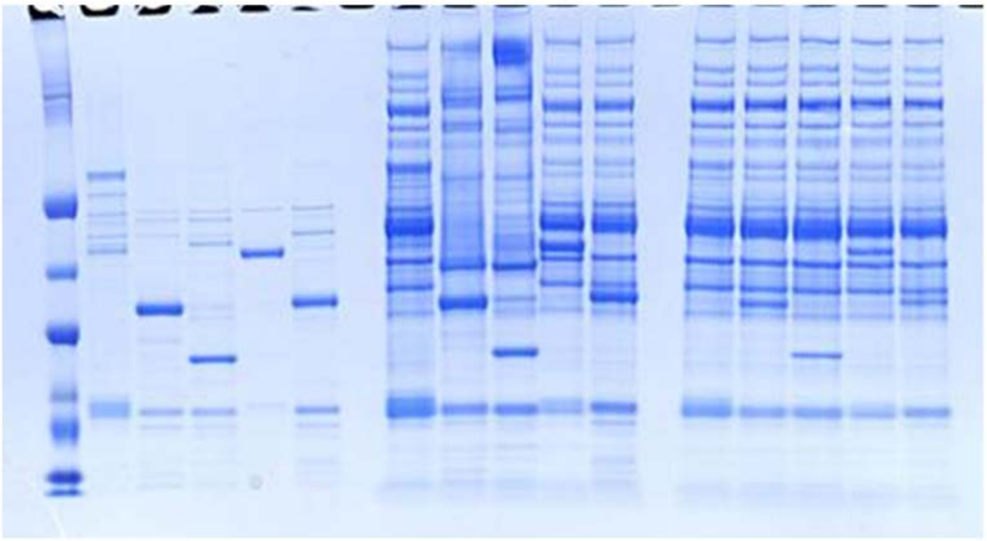

Supplement: Supplementary Information [file srep35391-s1.pdf]
